# Intelligent Biopolymer-Based Films for Food Quality Monitoring

**DOI:** 10.3390/polym18060694

**Published:** 2026-03-12

**Authors:** Diana-Ionela Dăescu, Diana-Maria Dreavă, Florina Stoica, Iulia Păușescu, Raluca Danciar, Gabriela Râpeanu, Anamaria Todea, Francisc Péter

**Affiliations:** 1 Faculty of Chemical Engineering, Biotechnology and Environmental Protection, Politehnica University of Timisoara, Vasile Pârvan Blv. 6, 300223 Timişoara, Romania; diana.daescu@student.upt.ro (D.-I.D.); diana.dreava@upt.ro (D.-M.D.); iulia.pausescu@upt.ro (I.P.); raluca.danciar@student.upt.ro (R.D.); francisc.peter@upt.ro (F.P.); 2 Department of Pedotechnics, Faculty of Agriculture, “Ion Ionescu de la Brad” University of Life Sciences, 3 Mihail Sadoveanu Alley, 700489 Iasi, Romania; florina.stoica@iuls.ro; 3 Department of Food Science, Food Engineering, Biotechnology and Aquaculture, Faculty of Food Science and Engineering, Dunărea de Jos University of Galati, 800201 Galați, Romania; gabriela.rapeanu@ugal.ro; 4 Research Institute for Renewable Energies (ICER), Politehnica University of Timisoara, Gavril Musicescu 138, 300501 Timişoara, Romania

**Keywords:** smart food packaging, biobased polymer film, flavylium dyes, pH-responsive, food freshness indicator

## Abstract

pH-responsive indicator films for intelligent food packaging applications are based on the embedding of a natural or synthetic dye in a polymeric substrate, preferably biobased and biodegradable. Although natural colorants like anthocyanins were extensively investigated in this respect, nature-inspired synthetic flavylium compounds could represent an alternative based on their higher stability. In this work, five novel synthetic 4′-aminoflavylium derivatives with different substitution patterns in the benzopyrylium core (compounds **1**–**5**) were synthesized and characterized. Polyvinyl alcohol (PVA), as well as chitosan–PVA and chitosan–starch blends, were used to prepare pH-responsive indicator films having inserted each of the synthesized flavylium dyes or a natural onion peel extract. The PVA films with compounds **1** and **3**, and the PVA–chitosan film with compound **1,** exhibited antioxidant activity, highlighting their potential for active packaging applications. All indicator films showed pH responsiveness in the range of 2 to 12 and were subsequently tested in contact with the packaging atmosphere or in direct contact with pork and fish meat, at different temperatures (4 °C, 20 °C, and 40 °C) for 24 h to assess their colorimetric response to progressive spoilage. Although the differences were small, the films with the 7-hydroxy-4′-aminoflavylium derivative exhibited the earliest and most intense color change during storage of meat, starting from direct contact at 4 °C for 24 h, being able to identify the initial stages of meat spoilage, while the performance of the dihydroxy-substituted derivative was attenuated by incorporation in polymer matrices. This behavior was comparable to that of onion peel extract, but the synthetic flavylium derivative was more stable. The results can provide new opportunities for intelligent food packaging applications using biopolymer indicator films with 4′-aminoflavylium derivatives.

## 1. Introduction

In the past years, the utilization of biobased and biodegradable plastics for food packaging has evolved from a niche environmental trend into a main category of sustainable packaging and a path toward the circular economy. Although petroleum-based synthetic polymers like polyethylene, polystyrene, or polyethylene terephthalate offer important advantages such as flexibility, easy processability, durability, excellent mechanical and barrier properties against moisture, oxygen, carbon dioxide, and contaminants, they currently face increasing environmental concerns due to greenhouse emissions, resource depletion, and climate change [1]. As plastic packaging consumption is expected to nearly triple by 2060 [2] and 60% of all plastic packaging is used for food and beverage packaging [3], these environmental issues will become increasingly challenging. Even though they have certain limitations, biobased and biodegradable polymers could be a sustainable alternative due to their renewability, biodegradability, recyclability, and low carbon footprint [2]. As a result, cellulose-, starch-, and polylactic acid-based biodegradable packaging films are already commercialized for various food items [4].

The global transport of food increases the need for effective freshness monitoring for producers, retailers, and consumers, as spoilage produces harmful compounds threatening human health. Traditional methods for detecting or evaluating food spoilage—physicochemical, microbial, and sensory—are slow, labor-intensive, and not real-time, requiring skilled operators and specialized labs [5]. Packaging has evolved from basic containment and storage using materials like glass, wood, and paper to sustainable, biodegradable solutions that extend shelf life, provide marketing information, and protect against environmental harm. Modern packaging technology focuses on active packaging, which releases antioxidants or antimicrobials from natural sources like plants and algae to preserve food, and intelligent packaging with sensors for real-time quality monitoring via color-changing indicators responsive to pH, moisture, or gases. Colorimetric indicators in packaging change color through chemical reactions or physical interactions with spoilage indicators like pH shifts, enabling easy freshness detection for meat or dairy without lab equipment. Natural pigments integrate well with biopolymers due to structural compatibility, supporting scalable, functional biodegradable systems [6].

There has been a growing interest in bio-based pH indicator systems including PVA/lignin/CMC double crosslinked matrices [7], Fe-CIT/PVA systems for shrimp monitoring [8], PVA/starch films incorporating anthocyanins from *Rhododendron arboreum* for chicken meat monitoring [9], chitosan/starch matrices containing amaranth flavonoids for beef spoilage detection [10], starch films with wild blackberry extract [11], PVA/starch matrices blended with propolis extract for milk monitoring [12], chitosan/PVA systems incorporating xanthylium dye [13], and chitosan, chitosan/PVA, PVA or PLA matrices blended with flavylium dye for pork and chicken meat spoilage indication [14].

Polyvinyl alcohol (PVA) is a water-soluble polymer recognized for its low cost, food compatibility, adjustable physicochemical properties, high transparency, and excellent film-forming characteristics, making it a viable candidate for substituting conventional fossil-derived plastic films in food packaging applications [7,15,16]. In addition, it is highly hydrophilic, and blending it with biopolymers can overcome some of its functional limitations [17]. In addition to its production from fossil feedstocks, PVA can be obtained from biobased sources. A US company, Bioplastics International, announced for the first time the manufacturing of PVA from sugar cane and alcohol [18]. Vinyl acetate (the starting material for PVA synthesis) from renewable sources is also commercially available [19].

Chitosan is a bio-based aminopolysaccharide polymer derived from partially deacetylated chitin obtained from crustacean shells, known for its low toxicity and cost. The amino functional groups of chitosan are responsible for its antibacterial activity, biodegradability, and biocompatibility. Although it showed good film-forming properties, its films generally present weak mechanical performance, which can be enhanced by crosslinking with materials such as polyvinyl alcohol (PVA), polylactic acid (PLA), glutaraldehyde, and carboxymethyl starch [20,21,22].

Starch, known for its wide availability, low cost, biodegradability, and good film-forming ability, is one of the most used materials for edible and biodegradable films. However, its hydrophilic nature causes high moisture sensitivity, weak mechanical strength, and brittleness, limiting practical applications. To overcome these limitations, it is frequently blended with other polymers, such as chitosan, gelatine, or modified through the incorporation of plasticizers and lipids, which improve structural stability, mechanical performance, and moisture resistance [21,23,24].

Anthocyanins are natural, water-soluble pigments widely found in flowers, fruits, leaves, and vegetables, responsible for a variety of intense colors. In plants, anthocyanins serve multiple roles, including acting as antioxidants, providing photoprotection, contributing to the defense mechanisms, and facilitating important biological functions [10,25,26,27]. These compounds demonstrated high pH sensitivity, making them excellent candidates for colorimetric sensing applications [20]. Despite their potential as pH indicators for monitoring meat freshness, anthocyanins present limitations such as low stability, susceptibility to decomposition, and limited sensitivity to subtle pH changes [27]. Synthetic flavylium derivatives can represent an alternative to anthocyanins for various applications [28].

This research aimed to synthesize and characterize five novel anthocyanin analog dyes and test their potential to monitor food quality in biopolymer films. The structures were confirmed by NMR and FT-IR analysis, followed by the investigation of the thermal and photochromic properties, as well as the antioxidant activities. The study also evaluated the performance of various biopolymer–flavylium dye compositions on pork and fish samples. Chitosan (CHIT), starch, and polyvinyl alcohol (PVA) blends were chosen as biobased and biodegradable polymeric matrices due to their favorable properties and their applicability in various sectors. The synthetic flavylium derivatives were investigated compared to onion peel extract, a natural product with demonstrated antioxidant potential [29], which was already reported as a potential freshness indicator in biopolymer films [30].

## 2. Materials and Methods

### 2.1. Materials

2,3-dihydroxybenzaldehyde (97%), 2,4-dihydroxybenzaldehyde (98%), 2,5-dihydroxybenzaldehyde (98%), 2,3,4-trihydroxybenzaldehyde (98%), and 2-hydroxy-5-methoxybenzaldehyde (98%) chitosan (low molecular weight, deacetylation ≥75%), trisodium phosphate (Na_3_PO_4_, 96%), citric acid (≥99.5%), 2,2-diphenyl-1-picrylhydrazyl (DPPH), polyvinyl alcohol (PVA, Mw 89,000–98,000 Da, >99% hydrolysed) and starch from potatoes (soluble) (Sigma Aldrich, Steinheim am Albuch, Germany. 4′-aminoacetophenone (99%) (Thermo Fisher Scientific, Heysham, UK). A red onion was purchased at a local market in Romania.

Sulfuric acid (H_2_SO_4_, 95–97%) and glacial acetic acid (CH_3_COOH, >99%) (CHIMREACTIV SRL (Bucharest, Romania). Boric acid (H_3_BO_3_, ≥99.5%) (VWR Life Science, Solon, OH, USA), methanol (MeOH, ≥99.98%), glycerol (87%), and diethyl ether (>99.98%) from VWR Chemicals BDH (Fontenay-sous-Bois, France). All reagents were used without any purification.

### 2.2. Methods

#### 2.2.1. Flavylium Dyes Syntheses

The flavylium dyes were prepared through a condensation reaction between 4′-aminoacetophenone and the proper benzaldehyde (2,3-dihydroxybenzaldehyde, 2,4-dihydroxybenzaldehyde, 2,5-dihydroxybenzaldehyde, 2,3,4-trihydroxybenzaldehyde, 2-hydroxy-5-methoxybenzaldehyde), by acid catalysis following a previously described procedure [31]. In all cases, the reaction resulted in the formation of a precipitate, which was isolated by filtration, washed with diethyl ether, and dried, yielding the hydrogensulfate flavylium salts.

#### 2.2.2. Onion Peel Extract

Red onion peel extract (**OPE**) was obtained using a conventional solvent extraction method (solid–liquid extraction), under agitation, as described by Vieira et al. [32] with a minor modification. The ratio between plant material and solvent was 1:15 (*w*/*v*). The solvent used was 70% ethanol, and each solvent extraction was acidified with glacial acetic acid at a ratio of 1:14 (acid/solvent, *v*/*v*). The pH values of the extracts after acid addition ranged from 2.12 to 2.67. Extractions were carried out at 25 °C for 1 h using an orbital shaker (SI-300R, Medline Scientific, Oxfordshire, UK) at 150 rpm. Subsequently, the samples were centrifuged (Hettich Universal 320R, Kirchlengern, Germany) for 10 min at 6000 rpm and 4 °C, and the supernatants were subjected to phytochemical characterization. The OPE was characterized in terms of total monomeric anthocyanin contents (milligrams cyanidin-3-glucoside (C3G) per gram of dry weight (dw)), total flavonoids (mg quercetin equivalents (QE) per g dw), total polyphenol contents (mg Gallic acid equivalents (GAE) per g dw), and DPPH radical scavenging activity (as µM Trolox/g dw and IC50, µg/mL).

The main characteristics of film formation are presented in Table 1. The assays were carried out as described in the literature [33].

#### 2.2.3. Film Preparation

##### PVA-Based Films (Abbreviated as PVA-)

Following the method described by Ma et al. with some adjustments, the first film was obtained by dissolving 1 g PVA and 10 mg of flavylium dye or 10 mg onion peel extract in 50 mL distilled water, followed by magnetic stirring at 70 °C until fully dissolved [34]. A similar procedure was used to prepare a dye-free film. Both solutions were subjected to ultrasonic treatment for CO_2_ removal, then carefully poured into Petri plates with a 15 cm diameter and dried in an oven for 72 h at 40 °C (≈4 mg dye/g dry film).

##### Chitosan–PVA-Based Films (Abbreviated as CHIT_PVA-)

Stock solutions of chitosan and PVA were prepared by dissolving 0.6 g chitosan in 30 mL of acetic acid 2% and 1.4 g PVA in 70 mL of water at 70 °C, respectively.

Following an adapted procedure described by Pereira et al., the film was prepared by mixing 15 mL of chitosan solution with 35 mL of PVA solution and adding 10 mg of flavylium dye or 10 mg of onion peel extract [35]. Similarly, a film without adding the dye was obtained. Both solutions were sonicated for 15 min to remove CO_2_, dispensed gradually into Petri plates with 15 cm diameter, and dried in the oven at 40 °C for 72 h (≈4.5 mg dye/g dry film).

##### Chitosan–Starch-Based Films (Abbreviated as CHIT_STARCH-)

A 5% starch solution was prepared by dissolving 2.5 g of starch in 50 mL of water. The dissolution was carried out under magnetic stirring at 80 °C for 30 min.

The blank solution was prepared by placing 0.5 g of chitosan into a beaker, followed by the addition of 25 mL of 2% acetic acid, 25 mL of 5% starch solution, and 0.2 mL of glycerol, as described by Bilgiç et al., with some adjustments [36]. The sample solution was prepared in the same manner as the blank solution, with the addition of 10 mg of the dye and 10 mg of onion peel extract.

The beakers containing the solutions were subjected to ultrasonic treatment for CO_2_ removal, carefully poured into Petri plates with a 15 cm diameter, then dried in an oven at 40 °C for 72 h (≈2.2 mg dye/g dry film).

#### 2.2.4. Evaluation of pH-Responsive Color Changes in Dyes and Films

Buffer solutions were prepared according to a previously described procedure [37], and the pH values were measured with a Mettler Toledo Seven Compact S210-K pH meter (Mettler Toledo, Columbus, OH, USA) at 25 °C.

For evaluating the photochromic properties of the dyes, a stock solution (50 mL, 10^−3^ M) was initially prepared. Samples were mixed in plastic cuvettes as follows: 200 µL dye stock solution + 2800 µL buffer solution of known pH. UV-Vis spectra were recorded at t = 0 and after defined intervals: 15 min, 30 min, 45 min, 1 h, 2 h, 3 h, 4 h, and 24 h.

Polymeric films (1 × 1 cm pieces) containing dye were immersed in 10 mL of buffer solutions with pH ranging from 2 to 12. After 2 h, the films were removed, gently wiped with filter paper, and their colorimetric response was evaluated by recording the UV-VIS absorption spectra. The UV-Vis spectra were acquired at 25 °C using an Agilent Cary 60 spectrophotometer (Agilent Technologies, Waldbronn, Germany).

#### 2.2.5. Antioxidant Activity/Capacity Evaluation for Dyes and Films

The antioxidant properties of flavylium salts were evaluated using UV-Vis spectroscopy and the stable DPPH radical, which exhibits maximum absorption at 517 nm. Samples were prepared in plastic cuvettes as follows: 2 mL methanol + 0.5 mL sample solution (concentration ranging from 6 × 10^−4^ to 9 × 10^−4^) + 0.5 mL DPPH solution (c = 10^−3^ M).

The antioxidant properties of the polymeric films containing dye were evaluated by cutting the films into 1 × 1 cm pieces, which were then immersed in 3 mL of methanol and subjected to ultrasonication following a previously reported procedure [38]. After 30 min, 2.5 mL of the resulting solutions were collected and mixed with 0.5 mL of 1 mM DPPH solution in plastic UV cuvettes. The samples were allowed to react at room temperature for 30 min before measuring the absorbance at 517 nm. A control solution was prepared by mixing 2.5 mL of methanol with 0.5 mL of DPPH. The antioxidant activity (as DPPH scavenging activity) was calculated using Equation (1), where A_DPPHi_ is the initial absorbance of DPPH and A_sample_ represents the absorbance of the sample:

(1) $$AA\left[\%\right]=\frac{{A_{DPPHi}-A}_{sample}}{A_{DPPHi}}\times 100$$

#### 2.2.6. Water Content, Swelling Capacity, and Solubility Evaluation

Water content, solubility, and swelling capacity were evaluated according to Zhong Y. and Silva M.A., with some adjustments. Polymeric films cut into 1 × 1 cm pieces were weighed (M_1_), dried at 105 °C until constant mass was achieved (M_2_), and then immersed in 30 mL distilled water in sealed vessels at room temperature for 24 h. The films were superficially dried with filter paper and placed in the oven at 105 °C until the mass was constant (M_3_). For swelling capacity, films (1 × 1 cm) were weighed (M_4_), immersed in 30 mL distilled water in sealed vessels at room temperature for 24 h, carefully wiped with filter paper, and weighed (M_5_) [39,40].

Three replicate measurements were performed for each film sample to determine the average value of the parameters. Water content, film solubility, and swelling capacity were calculated using Equations (2)–(4), respectively:

(2) $$Water\;content\;[\%]=\frac{M_{1}-M_{2}}{M_{1}}\times 100$$

(3) $$Water\;solubility\;[\%]=\frac{M_{3}-M_{2}}{M_{3}}\times 100$$

(4) $$Swelling\;capacity\left[\%\right]=\frac{M_{5}-M_{4}}{M_{4}}\times 100$$

#### 2.2.7. Testing of the Indicator Films on Food Products

The sensitivity of the indicator films for food quality changes was tested under direct contact and exposure to packaging atmosphere, respectively, using two types of food matrices: refrigerated fish and pork meat, at three temperatures: 4 °C, 20 °C, and 40 °C.

For direct contact, 1 × 1 cm film pieces were placed on 10 g meat samples, sealed in plastic foil, and stored at the specified temperatures for 24 h. For packaging atmosphere exposure, film pieces were placed in Petri dishes next to 10 g meat samples (without contact) and sealed with plastic foil under identical storage conditions.

UV-Vis spectra were recorded for all samples at t = 0 and after 24 h storage at the respective temperatures.

#### 2.2.8. Structural Analysis of the Synthesized Flavylium Compounds by NMR Spectroscopy

The synthesized dyes were analyzed by NMR spectroscopy using a Bruker Avance III spectrometer ((Bruker BioSpin GmbH, Rheinstetten, Germany) operating at 500 MHz (^1^H) and 125 MHz (^13^C) in DMSO-d_6_. 1D spectra recorded were ^1^H and ^13^C, while 2D spectra included COSY, HSQC, and HMBC. Chemical shifts are reported in ppm relative to tetramethylsilane (TMS), while the coupling constants are given in Hz.

#### 2.2.9. Characterization by Infrared Spectroscopy (ATR FT-IR)

FT-IR analyses of the synthesized compounds, onion peel extract, and the indicator films have been carried out using an IR Spirit Fourier Transform Infrared Spectrophotometer (Shimadzu, Kyoto, Japan). The measurements were recorded on a spectral domain of 4000–400 cm^−1^, and the spectrum was built after 64 co-added scans at a resolution of 2 cm^−1^.

#### 2.2.10. LC-MS Analysis

The LC-MS analyses were performed on a Microsaic 4000MiD mass spectrometer coupled to an Agilent 1100 HPLC, equipped with an ACE Excel 3 C18-AR column (150 × 3 mm, 3 µm). The mobile phase consisted of bidistilled water and methanol with 0.1% formic acid. The gradient program was as follows: 90% water (0 min) to 0% (15 min). The flow rate was 0.38 mL/min, with a column temperature of 35 °C. Mass spectra were acquired in positive ion mode over a *m*/*z* range of 100–800 using full scan acquisition, with a TIC voltage of 750 V. Injected sample concentrations were 300 µg/mL, with an injection volume of 5 µL.

#### 2.2.11. Thermogravimetric Analysis

Thermogravimetric analysis was performed using a TG 209 F1 Libra thermogravimetric analyzer (NETZSCH-Gerätebau GmbH, Selb, Germany) to obtain thermograms of the films and dye. Measurements were conducted under a nitrogen atmosphere from 20 to 600 °C at a heating rate of 10 K/min. Data were processed using Netzsch Proteus Thermal Analysis software version 6.1.0. (NETZSCH-Gerätebau GmbH, Selb, Germany).

## 3. Results and Discussion

### 3.1. Synthesis and Characterization of the Novel Flavylium Dyes

#### 3.1.1. Synthesis and Structural Characterization

**Figure 1:**
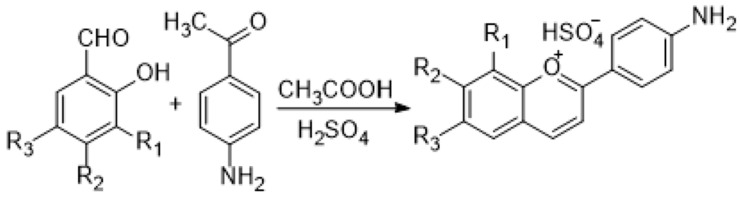
Reaction scheme of the synthesis of substituted 4′-aminoflavylium derivatives: (1) R_1_ = OH; R_2_ = R_3_ = H; (2) R_2_ = OH; R_1_ = R_3_ = H; (3) R_3_ = OH; R_1_ = R_2_ = H; (4) R_1_ = R_2_ = OH; R_3_ = H; (5) R_1_ = R_2_ = H; R_3_ = OCH_3._

This study reports the synthesis of five amino-substituted novel flavylium dyes (compounds **1**–**5**), with potential applications as sensors in smart food packages. These compounds belong to the class of 4′-aminoflavylium derivatives, substituted with hydroxy or methoxy groups in the positions 6, 7, or 8 of the benzopyrylium core. The syntheses were performed by aldol condensation of 4-aminoacetophenone with the appropriate substituted benzaldehydes. The reaction scheme is shown in Figure 1.

4′-amino flavylium derivatives are synthetic flavonoid compounds mimicking the natural anthocyanins but allowing tunable optoelectronic and biological properties. Pinto et al. showed that amino-substituted flavylium dyes are efficient in dye-sensitized solar cells due to the amino electron-donating group, which stabilizes the flavylium cation [41], while Correia et al. reported enhanced antibacterial effects of flavylium derivatives with amino or substituted amino groups in the C’4 position [42]. Our objective was to take advantage of the enhanced optoelectronic and biological properties of specifically substituted new 4′-aminoflavylium derivatives for their utilization in smart food packaging applications. The targeted compounds were obtained with excellent yields (between 76% and 82%) and high purity, without any need for further purification.

Structural analysis by ^1^H-NMR, ^13^C-NMR, LC-MS, and FT-IR demonstrated the formation of the targeted compounds in all cases based on the specific chemical shifts, molecular mass values, and absorption bands, respectively, presented below. The original spectra are provided as Supplementary Materials, including the numbering of the H and C atoms used for the NMR characterization (Figures S1–S3). Except for Compound **2** (considered as the most representative), the assignment of the FT-IR and NMR signals for the synthesized derivatives is also presented in the Supplementary Materials.

##### 8-Hydroxy-4′-aminoflavylium Hydrogensulfate (Compound **1**)

Brick-red: Precipitate, η = 81%; m.p. = 207–210 °C.

MS: Calculated molecular formula C_15_H_12_NO_2_^+^ 238.09, found [M]^+^ 238.4 (Figure S4a).

UV-Vis: λ_max_ = 506 nm (Figure S5a).

##### 7-Hydroxy-4′-aminoflavylium Hydrogfrensulfate (Compound **2**)

Orange precipitate, η = 82%; m.p. = 214–217 °C.

FT-IR (ATR) cm^−1^: 3293, 3257, asymmetric and symmetric stretching of the amino group and phenolic hydroxyl groups (ν_N–H_ and ν_O–H_); 3001, 2916, stretching of the C–H bonds in the phenyl and benzopyrylium rings (ν_C–H_ aromatic); 1635, 1614, skeletal stretching of the flavylium aromatic structure (ν_C=C_ and ν_C=O+_); 1579, 1472, stretching vibrations of the aromatic double bonds (ν_C=C arom_); 1417, in-plane bending of the hydroxyl groups (δ_O–H_); 1390, stretching of the C−N bond connecting the 4′-amino group (ν_C–N_); 1338, stretching of the phenolic C−O bonds (ν_C−O_); 1299, stretching of the C−O−C linkage within the pyrylium ring (ν_C−O−C_); 1258, stretching vibration which can be associated with the HSO4^−^ counterion (ν_SO4_); 842, 749, out-of-plane bending of aromatic C–H bonds, dependent on the substitution pattern (γ_C–H_).

^1^H-NMR (DMSO-d_6_, δ ppm): 9.93 (s, 1H, OH), 9.17 (s, 1H, H7), 8.12 (m, 2H, H12, H16), 7.88 (m, 4H, H3, H15), 6.63 (m, 1H, H6), 6.56 (dd, 1H, J= 5.70, 2.23 Hz, H2), 6.42 (dd, 1H, J = 8.50, 2.18 Hz, H8), 6.36 (s, 1H, H13).

^13^C-NMR (DMSO-d_6_, δ ppm): 165.14 (C9), 163.19 (C1), 135.84 (C5), 132.76 (C7), 130.17 (C3, C14), 129.74 (C2), 120.86 (C4), 117.66 (C13, C15), 115.15 (C11), 111.21 (C8), 108.60 (C6), 102.22 (C12, C16)

MS: Calculated molecular formula C_15_H_12_NO_2_^+^ 238.09, found [M]^+^ 238.4 (Figure S4b).

UV-Vis: λ_max_ = 524 nm (Figure S5b).

##### 6-Hydroxy-4′-aminoflavylium Hydrogensulfate (Compound **3**)

Burgundy precipitate, η = 80.4%; m.p. = 192–195 °C.

MS: Calculated molecular formula C_15_H_12_NO_2_^+^ 238.09, found [M]^+^ 238.4 (Figure S4c).

UV-Vis: λ_max_ = 518 nm (Figure S5c).

##### 7,8-Dihydroxy-4′-aminoflavylium Hydrogensulfate (Compound **4**)

Burgundy precipitate, η = 75.86%; m.p. = 174–177 °C.

MS: Calculated molecular formula C_15_H_12_NO_3_^+^ 254.08, found [M]^+^ 254.6 (Figure S4d).

UV-Vis: λ_max_ = 515 nm (Figure S5d).

##### 6-Methoxy-4′-aminoflavylium Hydrogensulfate (Compound **5**)

Brown precipitate, η = 79.5%; m.p. = 182–185 °C.

MS: Calculated molecular formula C_16_H_14_NO_2_^+^ 252.10, found [M]^+^ 252.6 (Figure S4e).

UV-Vis: λ_max_ = 510 nm (Figure S5e).

#### 3.1.2. Thermal Analysis

The thermal stability of the synthesized flavylium derivatives and onion peel extract was evaluated by thermogravimetric analysis, and the corresponding weight losses at selected temperature ranges are presented in Table 2. All samples exhibited an initial mass loss up to 16% below 200 °C, which can be attributed to the removal of adsorbed moisture. The weight loss increased substantially, especially upon heating to 300 °C, particularly for compounds **4** and **5** and **OPE**. At 600 °C, compound **1** showed the highest thermal stability (44.74%), while compound **5** presented the highest degradation (60.08%). The thermograms are presented in Figure S6.

#### 3.1.3. pH-Dependent Photochromic Properties

The pH-dependent photochromic behavior of flavylium dyes and onion peel extract was evaluated by UV-Vis spectroscopy. The presence of multiple chemical species at different pH values is evidenced by the overlaid collected UV-Vis spectra shown in Figures S7–S12. Color changes induced by pH variations were monitored over time to evaluate the stability of the different species.

Based on the available data, the synthesized compounds belong to the flavylium dye family, which exhibit photochromism through reaction pathways similar to those described for classical flavylium systems [13,43,44].

The photochromic behavior of the synthesized 4′-aminoflavylium derivatives is governed by electronic effects induced by the nature and position of substituents on the benzopyrylium core. The presence of an electron-donating amino group at the 4′ position increases charge delocalization throughout the conjugated system. This stabilizes the flavylium cation and shifts the absorption maximum toward longer wavelengths, which is a characteristic that sets these derivatives apart from natural anthocyanins. This electronic stabilization improves color persistence and reversibility under pH variation, both of which are essential features for intelligent food packaging applications.

Among the selected derivatives, the compound with a hydroxy group at position 7 of the benzopyrylium core showed the most pronounced, visually distinguishable pH-dependent color changes in solution and in biopolymer matrices. Structurally, substitution at position 7 promotes π-electron delocalization between the phenolic oxygen and the positively charged pyrylium ring. This facilitates the formation of quinoidal base species under mildly alkaline conditions. This structural arrangement lowers the energetic barrier for proton transfer reactions, resulting in a more sensitive and reversible chromatic response to the small pH variations associated with the early stages of food spoilage.

In contrast, the 6-hydroxy-substituted derivative exhibited a moderate chromatic response despite its good thermal and photochemical stability. The hydroxy group in position 6 engages in intramolecular hydrogen bonding with the adjacent oxygen atoms of the pyrylium ring. This leads to partial rigidification of the molecular framework. While this interaction enhances molecular stability, it also reduces the accessibility of the flavylium cation to hydration and ring-opening reactions. This decreases the system’s sensitivity to pH-induced structural transformations.

The lower sensitivity observed in the 8-hydroxy-substituted flavylium derivative is due to steric and electronic constraints associated with substitution at this position. The 8-hydroxy group contributes less effectively to conjugation with the flavylium core, which limits charge delocalization and reduces stabilization of the colored quinoidal species. Consequently, the resulting color transitions are less intense and less suitable for clear visual indication in practical packaging scenarios.

#### 3.1.4. Antioxidant Activity Determination

The antioxidant activity of the synthesized compounds was evaluated using the DPPH radical scavenging assay at different concentrations, expressed as IC_50_ values, representing the specific concentration of the compound required to scavenge 50% of free radicals in a test system. These values were calculated using an online resource, IC50 Calculator [45].

It can be observed from Table 3 that the lowest IC_50_ value (101.38 µg/mL) was obtained for compound **4**, followed by compound **3** (115.36 µg/mL). Compound **5** exhibited a considerably lower activity, with an IC_50_ of 301.17 µg/mL. It can be concluded that the synthesized compounds exhibited moderate-to-weak antioxidant activity, depending on their structure. As expected, the presence of phenolic hydroxy groups resulted in higher IC_50_ values compared to the methoxy group (in compound **5**), showing the highest value for the dihydroxy-substituted 4′-aminoflavylium derivative (compound **4**). At the same time, the position of the hydroxy group is also important. Although not being strong antioxidants, the inclusion of these compounds in food packages could also contribute to maintaining the freshness of the product.

Although the dihydroxy-substituted derivative exhibited the highest antioxidant activity among the synthesized compounds, its performance as a colorimetric indicator was comparatively attenuated when incorporated into polymeric films. The presence of two adjacent phenolic hydroxy groups increases the propensity for intermolecular hydrogen bonding and aggregation within the polymer matrix. These interactions can restrict molecular mobility and hinder the dynamic structural rearrangements required for efficient pH-responsive color transitions, highlighting the trade-off between antioxidant capacity and indicator performance.

Replacing the phenolic hydroxy group with a methoxy substituent resulted in a significant decrease in antioxidant activity and pH sensitivity. Unlike hydroxyl groups, methoxy substituents do not participate in proton exchange reactions and have a weaker electron-donating effect on the flavylium core. Consequently, quinoidal species are less stable, leading to diminished chromatic responsiveness under conditions relevant to food spoilage monitoring.

### 3.2. Characterization of the Polymeric Films with Embedded Flavylium Dye

Bio-based and biodegradable PVA, chitosan–PVA, and chitosan–starch polymeric films with embedded flavylium dye were prepared for every synthesized compound, as presented in the methods Section 2.2. FT-IR and UV-Vis spectroscopy were used to demonstrate the successful incorporation of the flavylium derivative in the polymer matrix, while the thermal stability was assessed by TGA. The most important characteristic of these polymeric films, which can give them applicability for the identification of food freshness, is the sensitivity to pH changes, evaluated by their UV-Vis behavior in the 2–12 pH range. At the same time, the interaction with water represents an essential characteristic that strongly influences the utilization limits, particularly during the storage of food products. All these investigations were also accomplished for an onion peel extract, a natural anthocyanin-containing product, for comparison. Ultimately, the capability of these embedded polymeric films to function as freshness indicators was tested in contact with the packaging atmosphere or direct contact with pork and fish meat at different temperatures.

The flavylium dyes were successfully retained within the PVA, chitosan–PVA, and chitosan–starch matrices after solvent evaporation. This retention is attributed to a combination of physical entrapment during solvent evaporation and stabilization through hydrogen bonding. The hydroxyl groups of PVA and starch, together with the hydroxyl and amino groups of chitosan, provide multiple interaction sites for the dye, enabling multiple hydrogen bonding interactions. The absence of new absorption bands in the FTIR spectra indicates that no covalent bond formation occurs, and that dye retention results from physical entrapment and non-covalent intermolecular interactions.

#### 3.2.1. FT-IR Spectroscopy

FT-IR spectroscopy was used to identify the main functional groups of the polymers used for the preparation of the bio-based polymeric films, PVA, chitosan, and starch, and to verify the incorporation of the dyes in the polymer matrices. The successful embedding of the flavylium derivatives was confirmed by the recorded FT-IR spectra for all polymer films. The spectra are included in the Supplementary Materials (Figures S13–S18).

**Figure 2:**
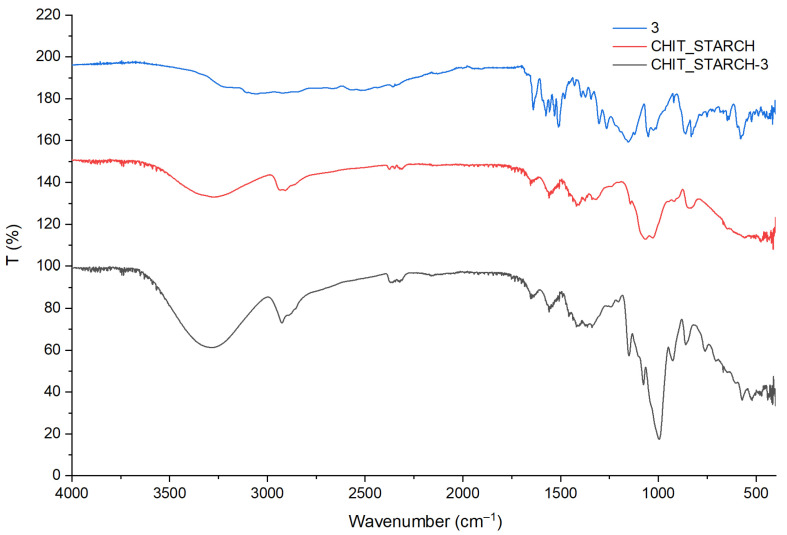
The FT-IR spectra of compound **3** (blue), chitosan–starch film (control, red), and chitosan–starch-**3** film (black).

The films obtained with compound **3** (6-hydroxy-4′-aminoflavylium hydrogensulfate) and PVA–starch polymer matrix were selected to illustrate in Figure 2 the effectiveness of FT-IR spectroscopy for the claimed purpose.

The chemical structures of chitosan and starch contain several identical functional groups (C–H, O–H, C–O); therefore, their vibrational bands are mostly overlapping. The presence of the amino/acetamide group in the chitosan molecule is the main difference. The characteristic bands of the control chitosan–starch film were assigned as follows: 3288 cm^−1^ (ν_OH_), 2925 cm^−1^ ($\nu_{{CH}_{2}}^{as}$), 1648 cm^−1^ (ν_C=O–_amide I-chitosan), 1557 cm^−1^ ($\delta_{{NH}_{2}}$ + ν_C–N_), 1145 cm^−1^ ($\nu_{COC}^{s})$, 1029 cm^−1^ ($\nu_{COC}^{as}$).

The characteristic FT-IR absorption bands of compound **3** were assigned previously in Section 3.1.1. In the spectrum of the embedded polymeric film (CHIT_STARCH-**3** in Figure 2), several bands are overlapping with those of the support matrix, particularly in the higher frequency region, but there are also specific bands which can be identified as originating from the flavylium derivative, mainly in the skeletal and substituted aromatic ring vibration domains, at 1405 cm^−1^ (the stretching vibrations of the aromatic double bonds, ν_C=C arom_), 1237 cm^−1^ (stretching of the C−N bond connecting the 4′-amino group, ν_C–N_), 1152 cm^−1^ and 1074 cm^−1^ (asymmetric and symmetric stretching vibration which can be associated with the HSO_4_^−^ counterion, ν_SO4_), and 758 cm^−1^ (out-of-plane bending of C–H bonds in monosubstituted aromatic rings, γ_C–H_). An increase in intensity of the broad band at 3288 cm^−1^ and in the region around 1000 cm^−1^ suggests the involvement of hydroxyl groups in hydrogen-bonding interactions between the flavylium dye and the polysaccharide matrix. In contrast, only minor changes were observed in the amide region (1648 cm^−1^), indicating that the amino group is not significantly involved. No new absorption bands were detected, supporting the absence of covalent bond formation.

#### 3.2.2. UV-Vis Spectroscopy

**Figure 3:**
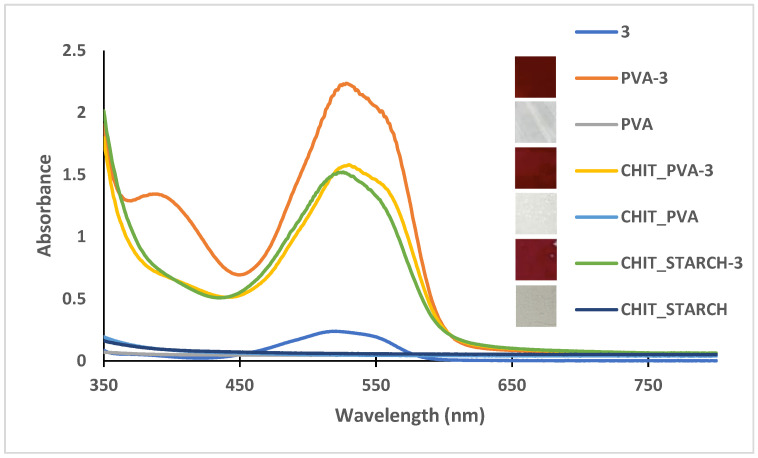
UV-Vis spectra of bio-based chitosan, chitosan–PVA, and chitosan–starch polymeric films (with and without compound **3**), compared to the spectrum of compound **3**.

The successful incorporation of the dye into the polymer matrices, as well as the transparency of the films, was confirmed by UV-Vis spectroscopy. The films prepared as described in the methods part were dried and analyzed under the same conditions as the corresponding control films, without dye. As shown in Table 4 and Figures S19–S23 (Supplementary Materials), most of the bio-based polymer films containing dye exhibited distinct absorption maxima in the visible region, except for PVA-**2** and chitosan–starch-**4**, whereas the control films displayed no significant absorption. These results are illustrated in Figure 3, presenting the UV-Vis spectra and the photographic images of the films prepared with the flavylium compound **3**, compared to the control films. The films with onion peel extract also showed characteristic absorption maxima in the visible region (Table 4), which can recommend them for inclusion in smart packaging materials.

#### 3.2.3. Thermal Analysis

The thermal behavior of the bio-based polymeric films was investigated using thermogravimetric analysis to determine their stability and characteristic decomposition temperatures.

In Figures S24–S29, the thermograms of all obtained polymeric blends and dyes are presented. It can be observed that PVA blank films presented three stages of weight loss, while chitosan–PVA and chitosan–starch blank films showed two stages.

PVA-**1**, PVA-**3**, PVA-**4**, chitosan–PVA-**2**, chitosan–PVA-**3**, chitosan–PVA-**4**, chitosan–PVA-**5**, PVA–OPE, and chitosan–PVA–OPE showed three stages of weight loss, PVA-**2** and PVA-**5** films presented four stages, while for all chitosan–starch films with the incorporated dyes and chitosan–PVA-**1** film, the weight loss occurred in two stages.

In the temperature range of 25–200 °C, the weight loss is attributed to the loss of surface moisture and intrinsic water from the polymeric films with onsets at 83.8 °C (PVA-**1** film), 72.4 °C (chitosan–PVA-**1** film), and 73.1 °C (chitosan–starch-**1** film); 85.9 °C (PVA-**2** film), 83.9 °C, 180.1 °C (chitosan–PVA-**2** film), and 62.4 °C (chitosan–starch-**2** film); 79.2 °C (PVA-**3**), 77.7 °C (chitosan–PVA-**3**), and 67.9 °C (chitosan–starch-**3**); 83.8 °C (PVA-**4**), 78.5 °C, 173.4 °C (chitosan–PVA-**4**), and 55.4 °C (chitosan–starch-**4** film); 81 °C (PVA-**5** film), 78.1 °C, 168.2 °C (chitosan–PVA-**5** film), and 67.6 °C (chitosan–starch-**5** film); 69.5 °C (PVA–**OPE** film), 76.8 °C, 170.4 °C (chitosan–PVA–**OPE** film), 74.2 °C (chitosan–starch-**OPE** film).

As can be observed in Tables S1–S6, the most important mass loss, which involved thermal degradation and decomposition, occurred in the temperature range of 200–400 °C, with onsets between 216.5 °C and 352.6 °C.

#### 3.2.4. pH Responsive Color Changes in the Indicator Polymer Films

The pH sensitivity of the films with incorporated dyes was evaluated by immersing the samples in 10 mL of buffer solutions covering a pH range from 2 to 12. After 2 h of immersion, the UV-Vis spectra of the films were recorded to assess shifts in absorption maxima and corresponding color changes.

**Figure 4:**
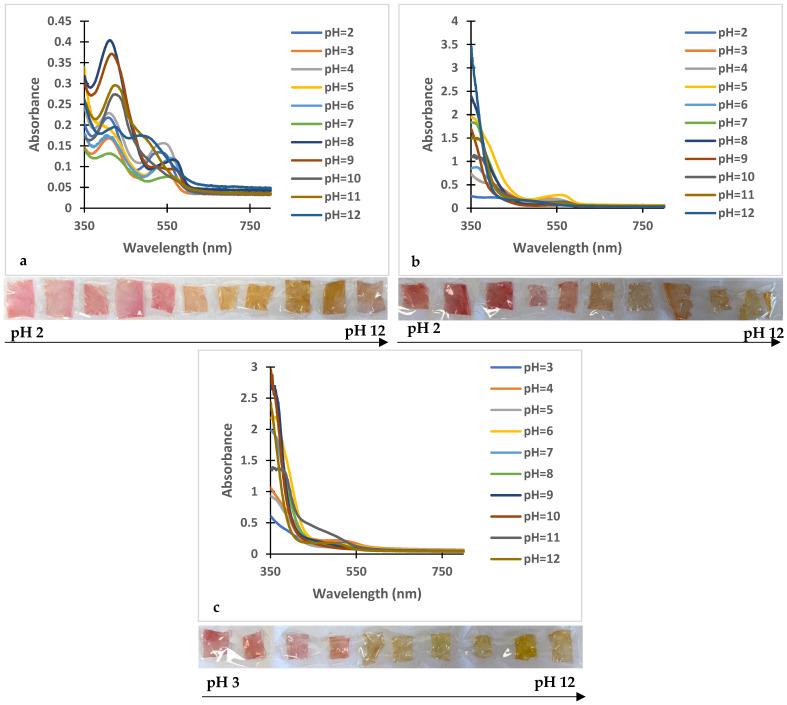
UV-Vis spectra of (**a**) PVA-**2** film; (**b**) chitosan–PVA-**2** film; (**c**) chitosan–starch-**2** film after 2 h in buffer solutions, and color change in the pH-responsive polymeric films.

The reduced structural compactness of the chitosan–starch matrix relative to the chitosan–PVA matrix led to the degradation of all chitosan–starch-based films at pH 2. With this exception, all films showed pH-dependent color changes, which can make them suitable for utilization as pH-responsive indicator films. The specific results for the films obtained with compound **2** are presented in Figure 4, while the Supplementary Material encompasses the behavior of the other pH-responsive four flavylium derivatives.

**Compound 1**: In the case of PVA films, small changes in the absorption maxima were observed at pH 9–10 and 11–12 (Figure S30a). Chitosan–PVA films displayed macroscopically visible color changes in the following pH intervals: 5–6, 8–9, and 11–12 (Figure S30b). For chitosan–starch films, four color transition stages were observed through pH ranges of 3–4, 6–7, 8–9, and 11–12 (Figure S30c, Supplementary Materials).

**Compound 2**: Color changes were observed in PVA films at pH values of 4–5, 6–7, 9–10, and 11–12. Chitosan–PVA films presented four stages of transition at pH 5–6, 7–8, 9–10, and 11–12, while chitosan–starch films presented visible changes in the pH ranges: 6–7, 10–11, and 11–12 (Figure 4).

**Compound 3**: Small shifts in the absorption maxima were observed for PVA films at pH 9–10 and 11–12, which were macroscopically visible (Figure S31a). Chitosan–PVA films displayed color changes in the pH ranges of 9–10, 10–11, and 11–12 (Figure S31b). Chitosan–starch films showed subtler changes, most pronounced at pH 5–6, 7–8, 8–9, and 9–10 (Figure S31c).

**Compound 4:** PVA films presented color changes in five pH ranges: 2–3, 4–5, 7–8, 9–10, and 10–11 (Figure S32a). Chitosan–PVA films displayed minor transitions, most evident at pH 4–5 and 9–10, while chitosan–starch films showed the most visible changes at pH 2–3 and 5–6 (Figure S32b,c).

**Compound 5:** PVA films showed changes in the pH ranges of 9–10 and 11–12 (Figure S33a). Chitosan–PVA films presented shifts in the absorption maxima in four pH ranges: 3–4, 9–10, 10–11, and 11–12 (Figure S33b). Chitosan–starch films displayed color variations at pH 6–7, 8–9, 10–11, and 11–12 (Figure S33c).

**Onion peel extract**: In the case of PVA, color transitions were observed at pH 9–10, 10–11, and 11–12 (Figure S34a). Chitosan–PVA films displayed four stages of transition at pH 6–7, 8–9, 10–11, and 11–12 (Figure S34b). Chitosan–starch films showed minor color changes at pH 6–7, 8–9, 9–10, and 11–12 (Figure S34c).

The polymeric environment modulates the structure–property relationships of the flavylium dyes. In PVA-based films, hydrogen bonding interactions between the polymer hydroxyl groups and the dye molecules contribute to good dispersion, though they may also partially dampen color intensity due to dilution effects. Chitosan–PVA blends offer a better balance of dye mobility and stabilization, enabling efficient proton exchange while maintaining structural integrity. In chitosan–starch matrices, stronger intermolecular interactions increase rigidity. Although this is beneficial for mechanical stability, it results in slower and less pronounced color transitions.

Importantly, the observed structure–property correlations directly translate into practical performance in food storage tests. The superior performance of the 7-hydroxy-4′-aminoflavylium derivative under refrigerated conditions shows that slight structural changes can greatly affect the sensitivity threshold of indicator films. This is particularly relevant for practical applications where the early detection of spoilage-related pH changes is critical for ensuring consumer safety and reducing food waste.

During the pH-response experiments, the migration behavior of the dyes was qualitatively evaluated by UV-Vis spectroscopy of the buffer solutions after the film immersion. Depending on the polymer matrix and the pH of the medium, partial dye release was observed for some film compositions. PVA-based films generally showed a higher tendency for dye migration, while matrices containing chitosan (chitosan–PVA and chitosan–starch) exhibited improved dye retention. This is due to stronger intermolecular interactions, such as hydrogen bonding and electrostatic effects. Migration was more pronounced at extreme pH values, where polymer swelling and structural changes are enhanced.

#### 3.2.5. Antioxidant Activity

Although the synthesized flavylium dyes showed moderate antioxidant activity, as discussed previously, their potential use in smart food packaging can be useful for preserving freshness. The investigation of the antioxidant properties was accomplished for all films prepared with the five flavylium salts and the onion peel extract.

As shown in Table 5, the antioxidant activity was significant only in PVA and PVA–chitosan films, with the highest DPPH radical inhibition values for PVA-1, chitosan–PVA-1, and PVA-3 (76.81%, 46.64%, and 45.87%, respectively). Blending of chitosan with starch resulted in almost complete loss of the antioxidant activity in the prepared films. The inclusion in polymeric films changed the hierarchy among the flavylium dyes, as the dihydroxy derivative 4 showed lower antioxidant activity compared to the best compound with a single hydroxy substituent in the benzopyrylium core (in this case, compound **1**). It should also be noticed that the natural onion peel extract exhibited lower antioxidant activity values in the PVA and PVA–CHIT matrices compared to the best synthetic compound **1**.

#### 3.2.6. Water Interaction Properties of the Investigated Polymeric Films

As shown in Table 6, the water content was less than 10% in the PVA–dye films and less than 13% in the chitosan–PVA–dye and chitosan–starch–dye films.

Films containing OPE generally displayed increased water content compared to those containing the flavylium dyes (13–18%), which can be attributed to the complex polyphenolic composition of the extract and its stronger interaction with water. PVA–dye and chitosan–PVA–dye films may be considered for applications requiring minimal water activity, while chitosan–starch–dye and OPE-containing blends may be suitable for applications where water retention is beneficial.

Regarding water solubility, PVA films displayed moderate solubility values, while the incorporation of chitosan led to an increase in solubility for chitosan–PVA films. Chitosan–starch films showed a higher water solubility, reflecting starch’s strong affinity for water. The swelling capacity of all films was significant, with values between 100% and 350% for PVA–dye matrix and non-measurable for the chitosan-containing films, making all of them suitable for detecting high-humidity conditions for food storage.

#### 3.2.7. Effectiveness of the Embedded Biopolymer Films for Monitoring the Freshness of Stored Meat Samples

The effectiveness of the biopolymer films with incorporated flavylium derivatives as intelligent food freshness indicator films was preliminarily evaluated using pork and fish samples, compared to the natural onion peel extract.

The films were kept in direct contact with meat and in contact with the packaging atmosphere at three different temperatures: 4 °C, 20 °C, and 40 °C. From considerations related to the size of the article, the results are presented in Table 7 for one selected flavylium derivative (compound **2**), and in the Supplementary Materials (Tables S7–S10) for the other compounds. The control films show in each case the initial color, before the interaction with the meat sample.

**Compound 1** (Figure S7): PVA films, which were in direct contact with pork and fish, showed significant color changes at all tested temperatures, while in the case of contact with the packaging atmosphere, the changes started to be more obvious at 20 °C and 40 °C. Chitosan–PVA films showed color transitions for both types of meat at all tested temperatures in direct contact and contact with the packaging atmosphere. Chitosan–starch films, which were in direct contact with meat, underwent a color intensity modification starting with 4 °C, while for the packaging atmosphere, the modifications were observed starting with 20 °C for pork, and no changes were observed for fish.

**Compound 2**: As shown in Table 7, PVA films exhibited significant modifications for all tested temperatures for both pork and fish in direct contact, while for contact with the packaging atmosphere, the most obvious changes were at 20 °C. Chitosan–PVA films responded with color transition starting with 4 °C for direct contact for both products, while for the packaging atmosphere, the most significant change was at 20 °C for pork, and 4 °C for fish. Chitosan–starch films showed for pork color transitions from 4 °C to 40 °C in direct contact, and for fish at 40 °C for both direct contact and contact with the packaging atmosphere.

**Compound 3** (Figure S8): PVA films which were in contact with the packaging atmosphere with pork and fish presented hypochromic shift regarding the intensity of absorption at 20 °C and 40 °C, while in the case of direct contact, the shifts started at 4 °C. Chitosan–PVA films started to show color transition at 4 °C in direct contact and 20 °C in contact with the packaging atmosphere on both pork and fish. Chitosan–starch films, which were in direct contact with pork and fish, underwent color intensity modifications from 4 °C to 40 °C, while in contact with the packaging atmosphere, the modifications were visible at 20 °C and 40 °C.

**Compound 4** (Figure S9): PVA films underwent a color modification only in direct contact with pork at 40 °C, while for fish, the color transition occurred at 40 °C for both testing conditions. Chitosan–PVA films tested on pork started to change their color at 20 °C in direct contact and 4 °C in contact with the packaging atmosphere, while for fish, the color changes occurred starting with 4 °C for both direct contact and contact with packaging atmosphere. Chitosan–starch films showed color changes starting at 20 °C for both testing conditions for pork, and 4 °C for fish in both testing conditions.

**Compound 5** (Figure S10): PVA films tested on pork showed visible color changes in direct contact with pork from 4 °C to 40 °C, while for films tested on fish in direct contact, the color changed at 20 °C and 40 °C, and 40 °C in contact with the packaging atmosphere. Chitosan–PVA films presented color transitions at all tested temperatures in direct contact, and 40 °C in contact with the packaging atmosphere for pork, while for fish, the most visible transitions were at 20 °C and 40 °C in direct contact, and 40 °C in contact with the packaging atmosphere. Chitosan–starch films responded with color modifications starting at 4 °C when tested in direct contact with both pork and fish, whereas for contact with the packaging atmosphere, the color transitions were visible at 20 °C and 40 °C.

**Onion peel extract** (Table 8): PVA films presented significant modifications at 20 °C and 40 °C in direct contact and at 40 °C in contact with the packaging atmosphere for both tested products. Chitosan–PVA films showed visible color changes at 20 °C and 40 °C in direct contact with pork and 40 °C in contact with the packaging atmosphere, while in direct contact with fish, the changes were visible at 40 °C for both testing conditions. Chitosan–starch films showed color transitions for both types of meat at all tested temperatures in direct contact and in contact with the packaging atmosphere.

These results are in accordance with the report of Torche et al., who used onion peel powder incorporated into cassava–starch films as a pH-sensitive indicator of minced beef freshness. These films showed gradual color shifts from light to dark brown across the pH range 1–13, but were investigated only for monitoring the storage under refrigerated conditions at 4 °C, the color change occurring between days 7 and 9 [46]. The utilization of anthocyanin extracts for intelligent food packaging in biodegradable polymers was reported by several groups, as reviewed by Janseerat et al. [47]. Chen et al. used 5–15% (wt.) blueberry anthocyanin-derived cyanidin in packaging films obtained from quaternary chitosan and gelatin for assessing the freshness of fresh shrimp at 20 °C for 48 h and found a correlation between the color change and the total volatile basic nitrogen content in the shrimp [48]. Although the utilization of natural anthocyanins looks very attractive, their large-scale extraction and purification could lead to increased costs, and their temperature and oxidative lability represent a well-known disadvantage. Onion peel extracts can represent a reliable solution, although the colorimetric scale is not so obvious. As for the synthetic flavylium compounds, they were only scarcely investigated as active compounds of freshness monitoring in intelligent biopolymer food packaging. Our research was more comprehensive compared to similar works, following the behavior of the films embedded with the new 4’-aminoflavylium derivatives at three different temperatures and two packaging circumstances, at direct and packaging atmosphere contact, demonstrating that the best compounds can lead to easily noticeable color changes during storage. Although several issues still need to be optimized, this approach could be a sustainable manufacturing solution.

## 4. Conclusions

Five new flavylium dyes were synthesized and comprehensively characterized. UV-Vis spectroscopy confirmed that the dyes exhibit pH-dependent photochromic behavior.

The flavylium dyes and a red onion peel extract were successfully incorporated into biodegradable structures based on PVA, chitosan–PVA, and chitosan–starch. Thermal properties of the films were evaluated by thermogravimetric analyses, revealing that the incorporation of the dyes or natural extract does not cause significant changes in behavior compared to the films without the dye.

The effect of these composite materials on the absorbance intensity was investigated. PVA films indicated the most visible changes for compounds **2** and **4**, while chitosan–PVA films showed a major change for compounds **1**, **5**, and **OPE**. Chitosan–starch films displayed the most evident changes for compounds **1**, **3**, **5**, and **OPE** in the pH range of 4 to 8, which is critical for their application as freshness indicators in intelligent packaging.

PVA-**1**, chitosan–PVA-**1**, and PVA-**3** films also presented antioxidant activity, making them candidates for active packaging. The other films presented an inability to release the dyes and the natural extract from the polymeric matrices.

The pH-responsive films were evaluated on refrigerated pork and fish, where distinct color changes were observed, demonstrating their suitability as indicators for intelligent packaging. Most of the films started to show significant changes at 20 °C in direct contact with the tested meats, while in contact with the packaging atmosphere, the most visible color changes were at 40 °C. The best results for detecting meat spoilage were given by 7-hydroxy-4′-aminoflavylium hydrogensulfate (compound **2**), which was able to detect incipient stages of food degradation for direct contact with the product, while in contact with the packaging atmosphere, almost all of the films could detect the pH changes starting with 20 °C. The films containing 7,8-dihydroxy-4′-aminoflavylium hydrogensulfate (compound **4**) were the most resistant when tested on food products, showing significant changes only for the chitosan–PVA and chitosan–starch matrices. These preliminary results confirm the suitability of the synthesized new 4′-aminoflavylium derivatives as indicators of food freshness in biodegradable polymeric films. Further, more detailed studies will give deeper insight into their efficient utilization in intelligent food packaging.

## Figures and Tables

**Table 1:** Phytochemical and antioxidant profile of red onion peel extract.

Total anthocyanins (mg C3G/g dw)	1.39 ± 0.04
Total flavonoids (mg QE/g dw)	85.15 ± 3.24
Total polyphenols (mg GAE/g dw)	74.82 ± 3.68
Antioxidant activity (µM Trolox/g dw)	27.94 ± 0.55
IC_50_ * (µg/mL)	70.71 ± 1.31

* IC_50_ (Inhibitory Concentration 50%) value represents the concentration of the OPE required to scavenge 50% of free radicals under the experimental conditions.

**Table 2:** Weight loss of compounds **1–5** and **onion peel extract** at different temperature ranges.

Compound	Weight Loss [%]				
	25–200 °C	25–300 °C	25–400 °C	25–500 °C	25–600 °C
**1**	6.73	15.51	32.69	39.07	44.74
**2**	12.01	23.62	45.16	50.57	55.07
**3**	9.04	20.06	39.17	46.22	51.63
**4**	10.89	27.04	42.64	49.82	56.14
**5**	6.01	31.32	42.97	50.25	60.08
OPE	15.50	30.62	40.26	48.92	54.64

**Table 3:** Calculated IC_50_ values for the synthesized compounds.

Compound	IC_50_ * (µg/mL)
**1**	174.20
**2**	194.48
**3**	115.36
**4**	101.38
**5**	301.17

* IC_50_ (inhibitory concentration 50%) value represents the concentration of the dye required to scavenge 50% of free radicals under the experimental conditions.

**Table 4:** UV-Vis absorption maxima of the bio-based dye–polymer matrices prepared with the synthesized flavylium derivatives and of the onion peel extract.

Compound	Maximum Wavelength of the Dye (nm)	Polymer Matrices	Maximum Wavelength of the Film (nm)
**1**	506	PVA-**1**	386
		CHIT_PVA-**1**	389
		CHIT_STARCH-**1**	377
**2**	524	PVA-**2**	-
		CHIT_PVA-**2**	368
		CHIT_STARCH-**2**	374
**3**	518	PVA-**3**	530
		CHIT_PVA-**3**	517
		CHIT_STARCH-**3**	525
**4**	515	PVA-**4**	374
		CHIT_PVA-**4**	410
		CHIT_STARCH-**4**	-
**5**	510	PVA-**5**	524
		CHIT_PVA-**5**	530
		CHIT_STARCH-**5**	520
OPE	362	PVA-**OPE**	379
		CHIT_PVA-**OPE**	375
		CHIT_STARCH-**OPE**	380

**Table 5:** Antioxidant activity of the polymeric films embedded with a flavylium dye or onion peel extract, respectively.

Polymeric Film	AA [%]
PVA-**1**	76.81 ± 15.60
CHIT_PVA-**1**	46.64 ± 6.49
CHIT_STARCH-**1**	4.03 ± 3.53
PVA-**2**	0.34 ± 0.78
CHIT_PVA-**2**	4.98 ± 3.44
CHIT_STARCH-**2**	1.78 ± 3.40
PVA-**3**	45.87 ± 6.75
CHIT_PVA-**3**	4.33 ± 1.60
CHIT_STARCH-**3**	2.09 ± 1
PVA-**4**	16.73 ± 3.06
CHIT_PVA-**4**	0.3 ± 0.52
CHIT_STARCH-**4**	2.53 ± 1.42
PVA-**5**	2.12 ± 6.25
CHIT_PVA-**5**	8.99 ± 2.18
CHIT_STARCH-**5**	-
PVA-**OPE**	12.12 ± 1.38
CHIT_PVA-**OPE**	9.65 ± 12.92
CHIT_STARCH-**OPE**	3.73 ± 0.93

**Table 6:** Water content, water solubility, and swelling capacity of the investigated polymeric films.

Polymeric Film	Water Content [%]	Water Solubility [%]	Swelling Capacity [%]
PVA-**1**	8.15 ± 1.50	11.68 ± 12.53	99.74 ± 37.19
CHIT_PVA-**1**	12.46 ± 13.72	15.21 ± 17.65	-
CHIT_STARCH-**1**	12.79 ± 5.72	31.79 ± 6.71	-
PVA-**2**	3.88 ± 1.10	23.26 ± 16.78	186.89 ± 10.14
CHIT_PVA-**2**	8.46 ± 0.25	14.15 ± 3.01	-
CHIT_STARCH-**2**	8.75 ± 0.75	28.12 ± 9.08	186.89 ± 10.14
PVA-**3**	4.35 ± 0.80	10.07 ± 6.99	349.81 ± 5.70
CHIT_PVA-**3**	12.50 ± 2.77	42.49 ± 12.36	-
CHIT_STARCH-**3**	11.41 ± 1.16	23.15 ± 1.91	-
PVA-**4**	7.37 ± 0.58	10.23 ± 1.36	135.92 ± 31.50
CHIT_PVA-**4**	8.77 ± 1.88	12.82 ± 3.43	-
CHIT_STARCH-**4**	9.46 ± 1.20	20.26 ± 1.65	-
PVA-**5**	3.78 ± 1.68	6.74 ± 0.69	342.16 ± 33.93
CHIT_PVA-**5**	8.04 ± 4.52	21.28 ± 3.22	-
CHIT_STARCH-**5**	10.60 ± 1.87	31.86 ± 10.95	-
PVA-**OPE**	4.81 ± 0.69	17.17 ± 1.40	174.53 ± 18.08
CHIT_PVA-**OPE**	10.07 ± 2.84	12.40 ± 1.84	-
CHIT_STARCH-**OPE**	12.82 ± 2.87	18.00 ± 3.29	-

**Table 7:** Color changes in the films with compound **2** were tested on meat samples at 4 °C, 20 °C, and 40 °C.

Meat	Control Film	Storage Condition	Temperature (°C)		
			4	20	40
Pork	PVA-**2**	atm contact	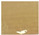	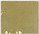	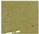
	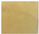	direct contact	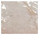	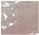	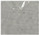
	CHIT_PVA-**2**	atm contact	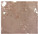	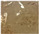	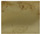
	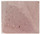	direct contact	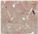	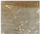	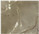
	CHIT_STARCH-**2**	atm contact	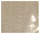	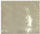	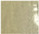
	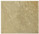	direct contact	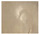	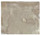	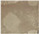
Fish	PVA-**2**	atm contact	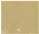	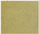	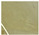
	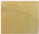	direct contact	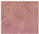	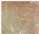	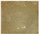
	CHIT_PVA-**2**	atm contact	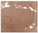	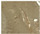	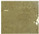
	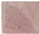	direct contact	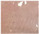	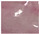	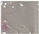
	CHIT_STARCH-**2**	atm contact	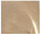	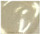	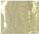
	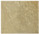	direct contact	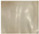	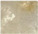	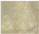

**Table 8:** Color changes in the films with onion peel extract (**OPE**), tested on meat samples at 4 °C, 20 °C, and 40 °C.

Meat	Control Film	Storage Condition	Temperature (°C)		
			4	20	40
Pork	PVA-**OPE**	atm contact	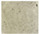	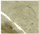	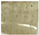
	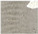	direct contact	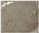	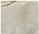	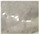
	CHIT_PVA-**OPE**	atm contact	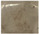	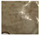	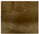
	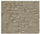	direct contact	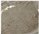	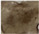	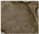
	CHIT_STARCH-**OPE**	atm contact	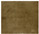	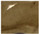	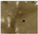
	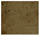	direct contact	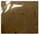	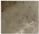	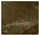
Fish	PVA-**OPE**	atm contact	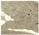	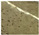	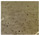
	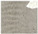	direct contact	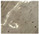	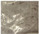	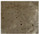
	CHIT_PVA-**OPE**	atm contact	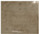	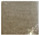	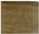
	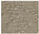	direct contact	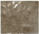	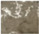	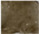
	CHIT_STARCH-**OPE**	atm contact	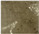	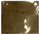	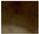
	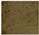	direct contact	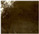	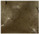	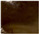

## Data Availability

The raw data supporting the conclusions of this article will be made available by the authors on request.

## Supplementary Materials

The following supporting information can be downloaded at: https://www.mdpi.com/article/10.3390/polym18060694/s1. Figure S1. FT-IR spectra of: (a) compound **1**; (b) compound **2**; (c) compound **3**; (d) compound **4**; (e) compound **5**; (f) onion peel extract. Figure S2. ^1^H NMR spectra of: (a) compound **1**; (b) compound **2**; (c) compound **3**; (d) compound **4**; (e) compound **5**. Figure S3. ^13^C NMR spectra of: (a) compound **1**; (b) compound **2**; (c) compound **3**; (d) compound **4**; (e) compound **5**. Figure S4. LC-MS spectra of: (a) compound **1**; (b) compound **2**; (c) compound **3**; (d) compound **4**; (e) compound **5**. Figure S5. UV-Vis spectrum of: (a) compound **1**; (b) compound **2**; (c) compound **3**; (d) compound **4**; (e) compound **5**; (f) onion peel extract. Figure S6. Thermogram of: (a) compound **1**; (b) compound **2**; (c) compound **3**; (d) compound **4**; (e) compound **5**; (f) onion peel extract. Figure S7. Absorption spectra of the species derived from compound **1** after (a) 0 min and (b) 24 h at different pH values. Figure S8. Absorption spectra of the species derived from compound **2** after (a) 0 min and (b) 24 h at different pH values. Figure S9. Absorption spectra of the species derived from compound **3** after (a) 0 min and (b) 24 h at different pH values. Figure S10. Absorption spectra of the species derived from compound **4** after (a) 0 min and (b) 24 h at different pH values. Figure S11. Absorption spectra of the species derived from compound **5** after (a) 0 min and (b) 24 h at different pH values. Figure S12. Absorption spectra of the species derived from OPE after (a) 0 min and (b) 24 h at different pH values. Figure S13. FTIR spectra of: (a) dye **1**, PVA (control), and PVA-**1**; (b) dye **1**, chitosan-PVA (control), and chitosan-PVA-**1**; (c) dye **1**, chitosan-starch (control), and chitosan-starch-**1**. Figure S14. FTIR spectra of: (a) dye **2**, PVA (control), and PVA-**2**; (b) dye **2**, chitosan-PVA (control), and chitosan-PVA-**2**; (c) dye **2**, chitosan-starch (control), and chitosan-starch-**2**. Figure S15. FTIR spectra of: (a) dye **3**, PVA (control), and PVA-**3**; (b) dye **3**, chitosan-PVA (control), and chitosan-PVA-**3**. Figure S16. FTIR spectra of: (a) dye **4**, PVA (control), and PVA-**4**; (b) dye **4**, chitosan-PVA (control), and chitosan-PVA-**4**; (c) **dye 4**, chitosan-starch (control), and chitosan-starch-**4**. Figure S17. FTIR spectra of: (a) dye **5**, PVA (control), and PVA-**5**; (b) dye **5**, chitosan-PVA (control), and chitosan-PVA-**5**; (c) dye **5**, chitosan-starch (control), and chitosan-starch-**5**. Figure S18. FTIR spectra of: (a) **OPE**, PVA (control), and PVA-**OPE**; (b) **OPE**, chitosan-PVA (control), and chitosan-PVA-**OPE**; (c) **OPE**, chitosan-starch (control), and chitosan-starch-**OPE**. Figure S19. UV-Vis spectra of bio-based polymeric films (control and with compound **1**). Figure S20. UV-Vis spectra of bio-based polymeric films (control and with compound **2**). Figure S21. UV-Vis spectra of bio-based polymeric films (control and with compound **4**). Figure S22. UV-Vis spectra of bio-based polymeric films (control and with compound **5**). Figure S23. UV-Vis spectra of bio-based polymeric films (control and with onion peel extract). Figure S24. Thermograms of: (**a**) PVA–**1** film (violet), PVA film (orange), and compound **1** (fuchsia); (**b**) chitosan–PVA–**1** film (light green), chitosan–PVA film (dark green), and compound **1** (fuchsia); (**c**) chitosan–starch–**1** film (blue), chitosan-starch film (red), and compound **1** (fuchsia). Figure S25. Thermograms of: (**a**) PVA–**2** film (brown), PVA film (orange), and compound **2** (green); (**b**) chitosan–PVA–**2** film (purple), chitosan–PVA film (azure), and compound **2** (green); (**c**) chitosan–starch–**2** film (purple), chitosan-starch film (red), and compound **2** (green). Figure S26. Thermograms of: (**a**) PVA–**3** film (pink), PVA film (orange), and compound **3** (purple); (**b**) chitosan–PVA–**3** film (azure), chitosan–PVA film (dark green), and compound **3** (purple); **c**) chitosan–starch–**3** film (green), chitosan-starch film (red), and compound **3** (purple). Figure S27. Thermograms of: (**a**) PVA–**4** film (brown), PVA film (orange), and compound **4** (purple); (**b**) chitosan–PVA–**4** film (pink), chitosan–PVA film (dark green), and compound **4** (purple); (**c**) chitosan–starch–**4** film (green), chitosan-starch film (red), and compound **4** (purple). Figure S28. Thermograms of: (**a**) PVA–**5** film (purple), PVA film (orange), and compound **5** (azure); (**b**) chitosan–PVA–**5** film (olive), chitosan–PVA film (dark green), and compound **5** (azure); (**c**) chitosan–starch–**5** film (grey), chitosan-starch film (red), and compound **5** (azure). Figure S29. Thermograms of: (**a**) PVA–**OPE** film (pink), PVA film (orange), and **OPE** (purple); (**b**) chitosan–PVA–**OPE** film (brown), chitosan–PVA film (dark green), and **OPE** (purple); (**c**) chitosan–starch–**OPE** film (olive), chitosan-starch film (red), and **OPE** (purple). Figure S30. UV-Vis spectra of: (a) PVA-**1** film; (b) chitosan-PVA-**1** film; (c) chitosan-starch-**1** film after 2h in buffer solutions. Figure S31. UV-Vis spectra of: (a) PVA-**3** film; (b) chitosan-PVA-**3** film; (c) chitosan-starch-**3** film after 2h in buffer solutions. Figure S32. UV-Vis spectra of: (a) PVA-**4** film; (b) chitosan-PVA-**4** film; (c) chitosan-starch-**4** film after 2h in buffer solutions. Figure S33. UV-Vis spectra of: (a) PVA-**5** film; (b) chitosan-PVA-**5** film; (c) chitosan-starch-**5** film after 2h in buffer solutions. Figure S34. UV-Vis spectra of: (a) PVA-**OPE** film; (b) chitosan-PVA-**OPE** film; (c) chitosan-starch-**OPE** film after 2h in buffer solutions. Table S1. Weight losses of the blank and compound **1** incorporated PVA, chitosan-PVA, chitosan-starch films and compound **1**. Table S2. Weight losses of the blank and compound **2** incorporated PVA, chitosan-PVA, chitosan-starch films and compound **2**. Table S3. Weight losses of the blank and compound **3** incorporated PVA, chitosan-PVA, chitosan-starch films and compound **3**. Table S4. Weight losses of the blank and compound **4** incorporated PVA, chitosan-PVA, chitosan-starch films and compound **4**. Table S5. Weight losses of the blank and compound **5** incorporated PVA, chitosan-PVA, chitosan-starch films and compound **5**. Table S6. Weight losses of the blank and **OPE** incorporated PVA, chitosan-PVA, chitosan-starch films and **OPE**. Table S7. Color changes of compound **1** incorporated films tested on meat at 4 °C, 20 °C, and 40 °C. Table S8. Color changes of compound **3** incorporated films tested on meat at 4 °C, 20 °C, and 40 °C. Table S9. Color changes of compound **4** incorporated films tested on meat at 4 °C, 20 °C, and 40 °C. Table S10. Color changes of compound **5** incorporated films tested on meat at 4 °C, 20 °C, and 40 °C.

## Nomenclature and Abbreviations

η	Yield
m.p.	Melting point
PVA	Polyvinyl alcohol
CHIT	Chitosan
OPE	Onion peel extract
C3G	Cyanidin-3-glucoside
QE	Quercetin equivalents
GAE	Gallic acid equivalents
dw	Dry weight
A_DPPHi_	Initial absorbance of DPPH
A_sample_	Absorbance of the sample
