# Supplementary material for: Intelligent Biopolymer-Based Films for Food Quality Monitoring
Source: Polymers (Basel). 2026 Mar 12;18(6):694. doi: 10.3390/polym18060694 (PMC13029925; doi:10.3390/polym18060694)
Supplement: Supplementary file 1 [file polymers-18-00694-s001.zip › polymers-4183876-supplementary.pdf]

Intelligent biopolymer-based films for food quality monitoring

Diana Dăescu [1], Diana-Maria Dreavă [1], Florina Stoica[3], Iulia Păușescu [1], Raluca Danciar [1], Gabriela Râpeanu[4], and Anamaria Todea [1*], Francisc Péter [1,2]

[1] Faculty of Chemical Engineering, Biotechnology and Environmental Protection, Politehnica University of Timisoara, Vasile Pârvan Blv.6, 300223 Timişoara, Romania
[2] Research Institute for Renewable Energies (ICER), Politehnica University of Timisoara, Gavril Musicescu 138, 300501 Timişoara, Romania
[3] Department of Pedotechnics, Faculty of Agriculture, "Ion Ionescu de la Brad" University of Life Sciences, 3 Mihail Sadoveanu Alley, 700489 Iasi, Romania; florina.stoica@iuls.ro
[4] Department of Food Science, Food Engineering, Biotechnology and Aquaculture, Faculty of Food Science and Engineering, Dunărea de Jos University of Galati, 800201, Galați, România; gabriela.rapeanu@ugal.ro
* Correspondence: anamaria.todea@upt.ro

Supplementary material

Contents

1. Compounds characterization 2

1.1. FT-IR characterization of flavylium dyes 2

1.2. NMR characterization of flavylium dyes 4

1.3. LC-MS 8

1.4. UV-Vis 9

1.5. Thermogravimetric characterization of the dyes and onion peel extract 10

1.6. pH-dependent photochromic properties 11

2. Polymeric films 14

2.1. FT-IR 14

2.2. UV-Vis spectroscopy 20

2.3. Thermal Analysis 21

2.4. Films' sensitivity to pH changes 25

2.5. Effectiveness of the embedded biopolymer films for monitoring the freshness of stored meat samples 30

1. Compounds characterization

1.1. FT-IR characterization of flavylium dyes

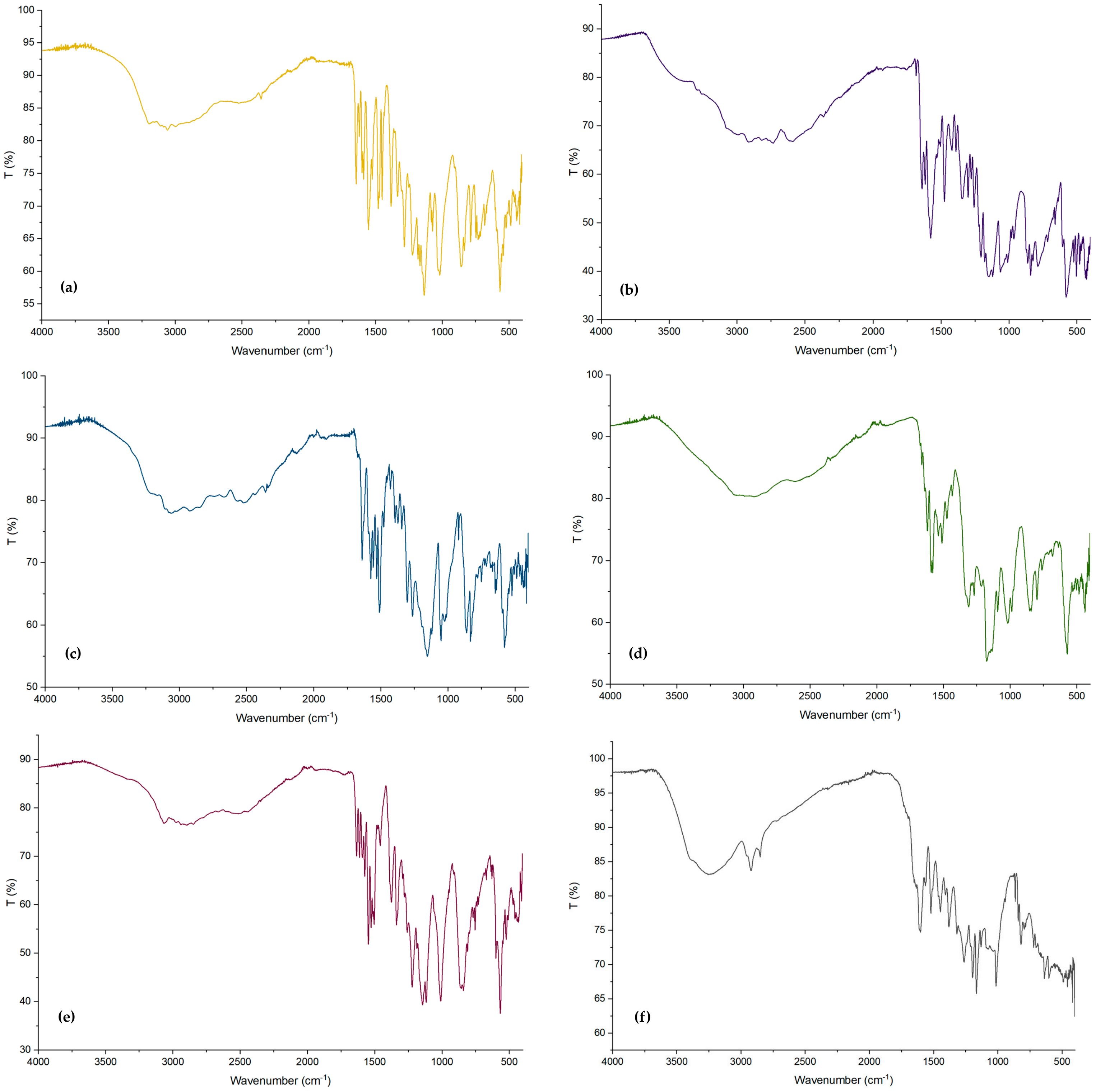

Figure S1. FT-IR spectra of: (a) **compound 1**; (b) **compound 2**; (c) **compound 3**; (d) **compound 4**; (e) **compound 5**; (f) **onion peel extract**.

Compound 1: FT-IR (ATR) cm^{-1}: 3195, stretching of the amino group and phenolic hydroxyl groups (ν_{N-H} and ν_{O-H}); 3064, 3003, stretching of the C−H bonds in the phenyl and benzopyrylium rings (ν_{C-H} aromatic); 2545, O-H stretching assigned to strong intermolecular hydrogen bonding in the bisulphate anion ($\nu_{O-H...O}$); 1648, skeletal stretching of the flavylium aromatic structure ($\nu_{C=O+}$); 1599, 1548, stretching vibrations of the aromatic double bonds ($\nu_{C=C}$ aromatic); 1477, in-plane bending of the hydroxyl groups (δ_{O-H}); 1452, stretching of the C−N bond connecting the 4′-amino group (ν_{C-N}); 1386, stretching of the phenolic C−O bonds (ν_{C-O}); 858, out-of-plane bending of aromatic C−H bonds, dependent on the substitution pattern (γ_{C-H}).

Compound 2 – in the main text

Compound 3: FT-IR (ATR) cm^{-1}: 3220, 3200, overlapping asymmetric and symmetric stretching of the amino group and phenolic hydroxyl groups (ν_{N-H} and ν_{O-H}); 3104, 3053, stretching of the C−H bonds in the phenyl and benzopyrylium rings (ν_{C-H} aromatic); 2530, 2437 O-H stretching assigned to strong intermolecular hydrogen bonding in the sulphate salt ($\nu_{O-H...O}$); 1637, skeletal stretching of the flavylium structure ($\nu_{C=O^+}$); 1576, 1556, skeletal stretching of the aromatic structure ($\nu_{C=C}$); 1532, 1512, stretching vibrations of the aromatic double bonds ($\nu_{C=C}$ aromatic); 1306, stretching of the phenolic C−O bond in position 6 (ν_{C-O}); 1267, stretching of the C−N bond connecting the 4′-amino group (ν_{C-N}); 1299, stretching of the C−O−C linkage within the pyrylium ring (ν_{C-O-C}); 1152, 1054, asymmetric and symmetric stretching vibration which can be associated with the HSO_4^- counterion (ν_{SO4}); 859, 830, out-of-plane bending of aromatic C−H bonds, dependent on the substitution pattern (γ_{C-H}).

Compound 4: FT-IR (ATR) cm^{-1}: 3059, 2908, stretching of the C−H bonds in the phenyl and benzopyrylium rings (ν_{C-H} aromatic); 2611, O-H stretching assigned to strong intermolecular hydrogen bonding in the sulphate salt and 7,8-dihydroxy groups ($\nu_{O-H...O}$); 1622, skeletal stretching of the flavylium structure ($\nu_{C=O^+}$); 1582, 1541, skeletal stretching of the aromatic ring ($\nu_{C=C}$); 1510, in plane amine scissoring (δ_{NH2}); 1477, stretching vibrations of the aromatic double bonds ($\nu_{C=C}$ aromatic); 1308, stretching of the phenolic C−O bonds (ν_{C-O}); 1271, stretching of the C−N bond connecting the 4`-amino group (ν_{C-N}); 1222, 1177, asymmetric stretching vibration which can be associated with the HSO4− counterion (ν_{asSO4}); 1094, stretching of the C−O−C linkage within the pyrylium ring (ν_{C-O-C}); 1011, 985, symmetric stretching vibration which can be associated with the $HSO4^-$ counterion (ν_{sSO4}); 844, out-of-plane bending of aromatic C−H bonds, dependent on the substitution pattern (γ_{C-H}).

Compound 5: FT-IR (ATR) cm^{-1}: 3350, N-H stretching in the 4′-amino group; 3059, stretching of the C−H bonds in the phenyl and benzopyrylium rings (ν_{C-H} aromatic); 2968, 2905, 2841, asymmetric and symmetric C-H stretching in the 6-methoxy group; 1632, skeletal stretching of the flavylium structure ($\nu_{C=O^+}$); 1611, 1589, skeletal stretching of the aromatic ring ($\nu_{C=C}$); 1545, in plane amine scissoring (δ_{NH2}); 1503, stretching vibrations of the aromatic double bonds ($\nu_{C=C}$ aromatic); 1460, C-H scissoring of the methoxy methyl group (δ_{C-H}); 1335, stretching of the phenolic C−O bonds (ν_{C-O}); 1218, stretching of the C−N bond connecting the 4′-amino group (ν_{C-N}); 1147, 1011, stretching vibrations which can be associated with the $HSO4^-$ counterion (ν_{sSO4}); 854, out-of-plane bending of aromatic C−H bonds, dependent on the substitution pattern (γ_{C-H}).

1.2. *NMR characterization of flavylium dyes*

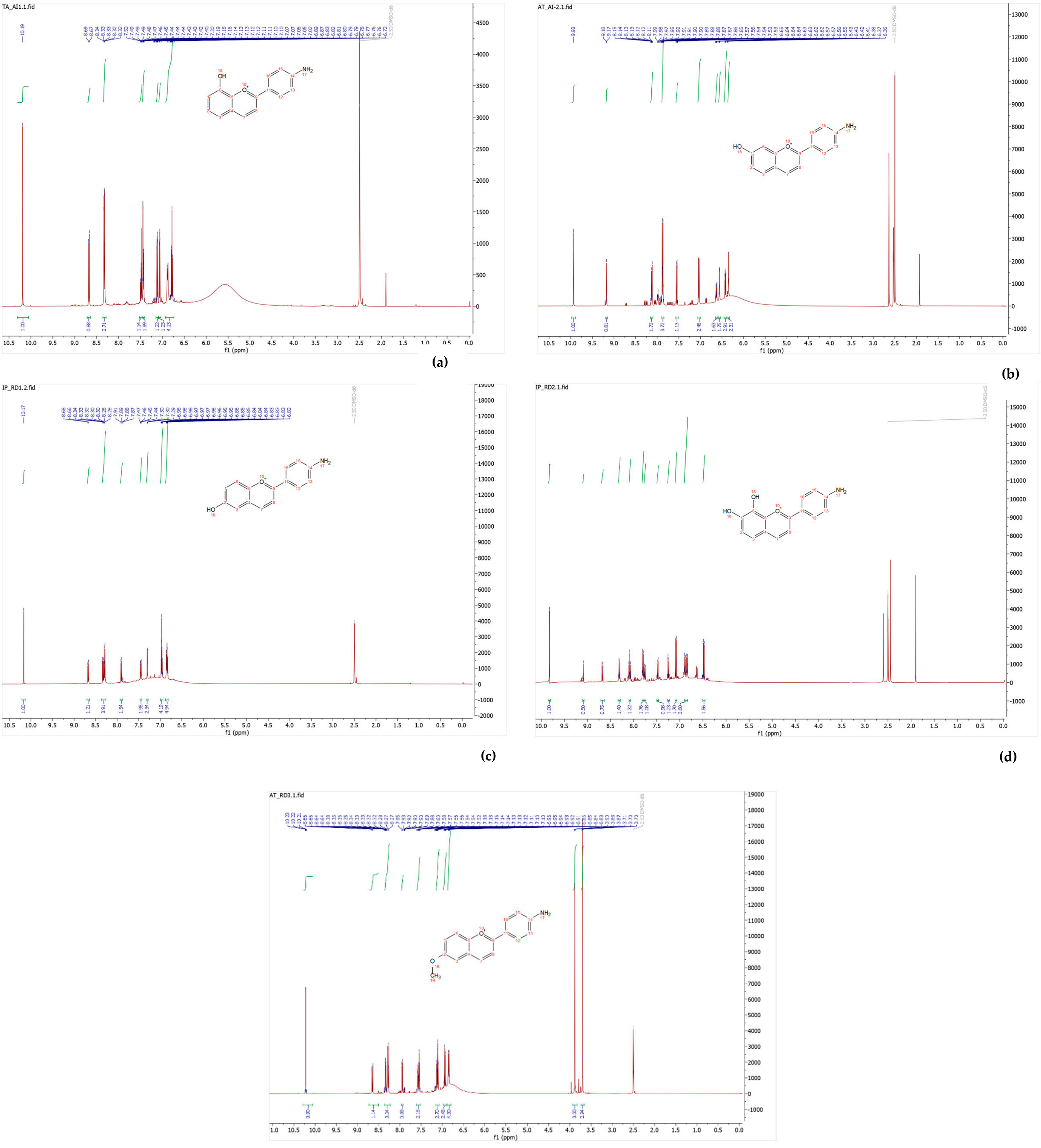

Figure S2. ^{1}H NMR spectra of: (a) **compound 1**; (b) **compound 2**; (c) **compound 3**; (d) **compound 4**; (e) **compound 5**.

Compound 1: ^{1}H-NMR (DMSO-d$_6$, δ ppm): 10.19 (s, 1H, H18), 8.68 (d, 1H, J=9.48 Hz, H7), 8.33 (dd, 2H, J= 9.41, 1.12 Hz, H2, H8), 7.49 (m, 1H, H16), 7.43 (m, 2H, H13, H15), 7.12 (dd, 1H, J= 7.84, 16.60 Hz, H12), 7.06 (dd, 1H, J= 7.78, 1.58 Hz, H1), 6.82 (m, 4H, overlapping aromatic signals including H3).

Compound 2: in the main text

Compound 3: ^{1}H-NMR (DMSO-d$_6$, δ ppm): 10.02 (s, 1H, H18), 8.67 (d, 1H, J = 9.49 Hz, H7), 8.31 (m, 2H, H6, H8), 7.90 (d, 2H, J = 9.14 Hz, H13, H15), 7.45 (dd, 1H, J= 9.13, 2.88 Hz, H12, H16), 7.30 (d, 1H, J= 2.87 Hz, H1), 6.84 (m, 1H, H3).

Compound 4: ^{1}H-NMR (DMSO-d$_6$, δ ppm): 9.82 (s, 1H, 19), 9.09 (d, 1H, H18), 8.68 (d, 1H, J = 9.22 Hz, H7) 8.68 (d, 1H, J = 9.22 Hz, H8), 8.31 (d, 1H, J = 8.84 Hz, H8), 8.09 (t, 1H, J = 8.78 Hz, H12), 7.79 (m, 2H, H3, H2), 7.25 (d, 1H, J = 8.61 Hz, H16), 7.08 (d, 1H, J = 8.56 Hz, H13), 6.86 (m, 3H, H15).

Compound 5: ^{1}H-NMR (DMSO-d$_6$, δ ppm): 10.22 (s, 1H, H17), 8.65 (m, 1H, H7), 8.33-8.27 (2d, 4H, J = 9.52, 9.13 Hz, H6, H8), 7.94 (m, 1H, H6), 7.56 (m, 2H, H13, H15), 7.11 (m, 3H, H12, H16), 6.94 (d, 2H, J= 8.80 Hz, H1), 6.85 (d, J = 8.91 Hz, 4H, H3), 3.88-3.70 (s, 3H, H19).

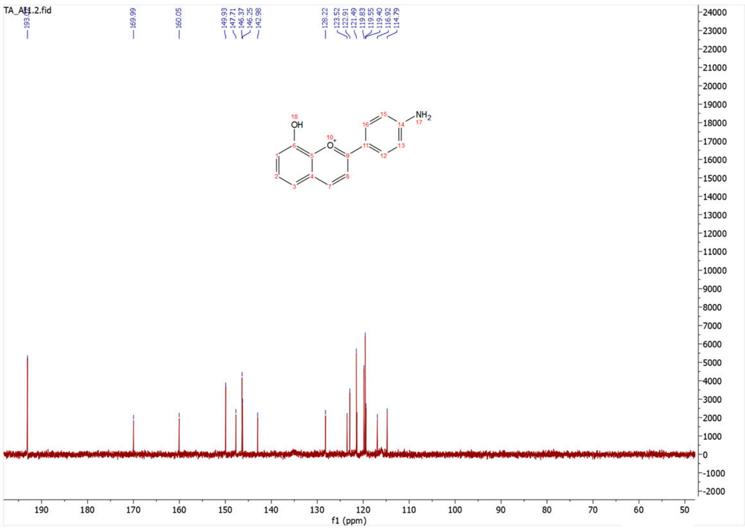

(a)

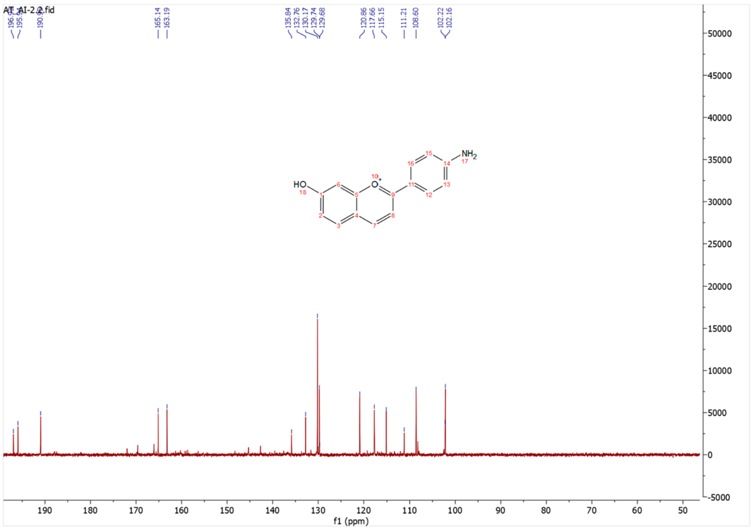

(b)

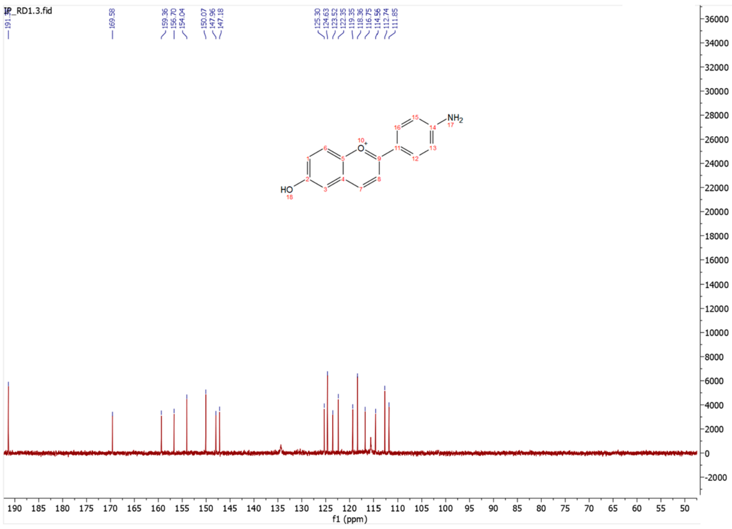

(c)

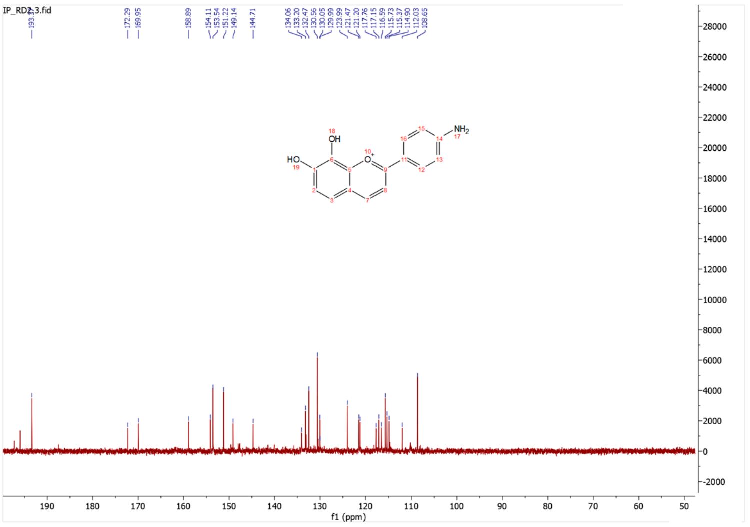

(d)

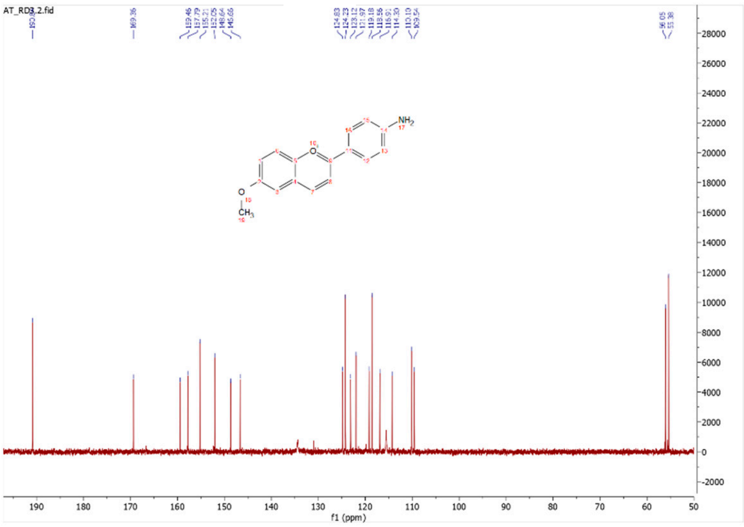

Figure S3. ^{13}C NMR spectra of: (a) **compound 1**; (b) **compound 2**; (c) **compound 3**; (d) **compound 4**; (e) **compound 5**.

Compound 1: ^{13}C-NMR (DMSO-d_6, δ ppm): 169.99 (C9), 160.05 (C7), 149.93 (C14), 147.71 (C5), 146.37 (C13), 146.25 (C15), 142.98 (C6), 128.22 (C1), 123.52 (C3), 122.91 (C4), 121.49 (C2), 119.83 (C11), 119.55 (C8), 119.40 (C12), 116.92 (C16).

Compound 2 – in the main text

Compound 3: ^{13}C-NMR (DMSO-d_6, δ ppm): 169.58 (C9), 159.36 (C2), 156.70 (C7), 154.04 (C5), 150.07 (C13), 147.57 (C15), 147.18 (C11), 125.30 (C1), 124.63 (C4), 123.52 (C6), 122.35 (C14), 119.35 (C8), 118.36 (C3), 116.75 (C12), 112.30 (C16).

Compound 4: ^{13}C-NMR (DMSO-d_6, δ ppm): 169.95 (C9), 158.89 (C1), 154.14 (C7), 153.54 (C13), 151.22 (C15), 149.14 (C14), 144.71 (C11), 133.20 (C6), 132.47 (C3), 129.99 (C2), 121.20 (C4), 116.59 (C5), 115.73 (C12), 115.37 (C16), 108.65 (C8).

Compound 5: ^{13}C-NMR (DMSO-d_6, δ ppm): 190.84 (C9), 159.46 (C11), 157.79 (C5), 155.21 (C14), 152.05 (C13), 148.64 (C12), 146.66 (C4), 124.83 (C15), 124.23 (C16), 123.12 (C1), 121.19 (C3), 119.18 (C6), 118.56-116.91 (C7), 114.30 (C8), 110.10 (C2), 56.05 (C19).

1.3. *LC-MS*

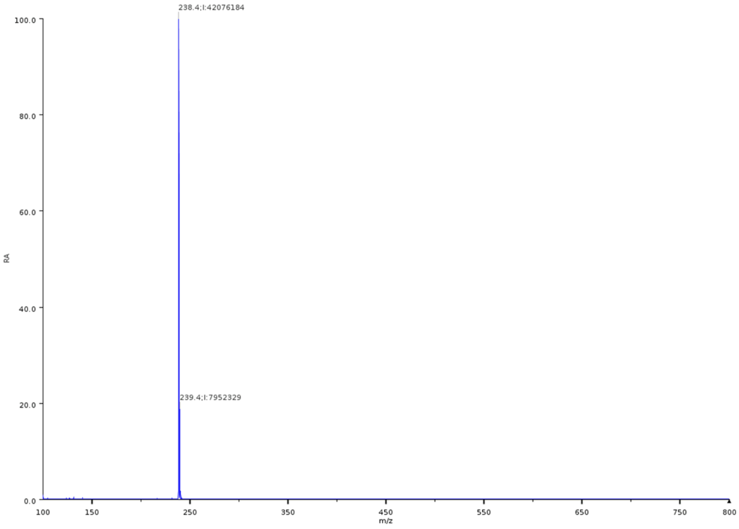

(a)

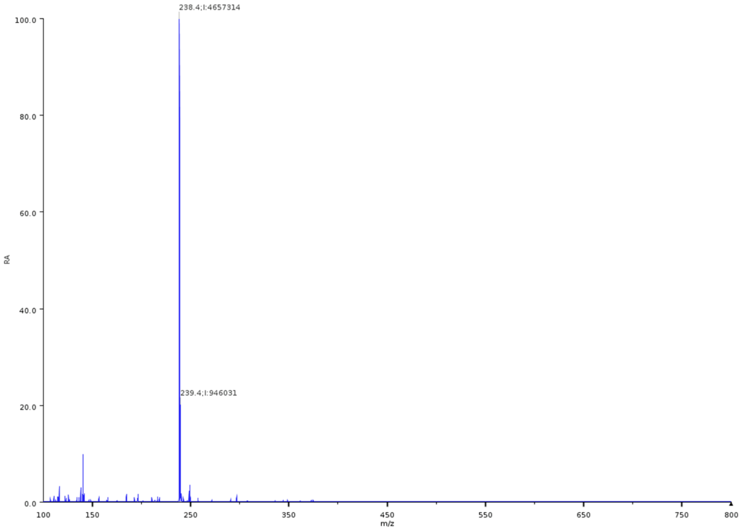

(b)

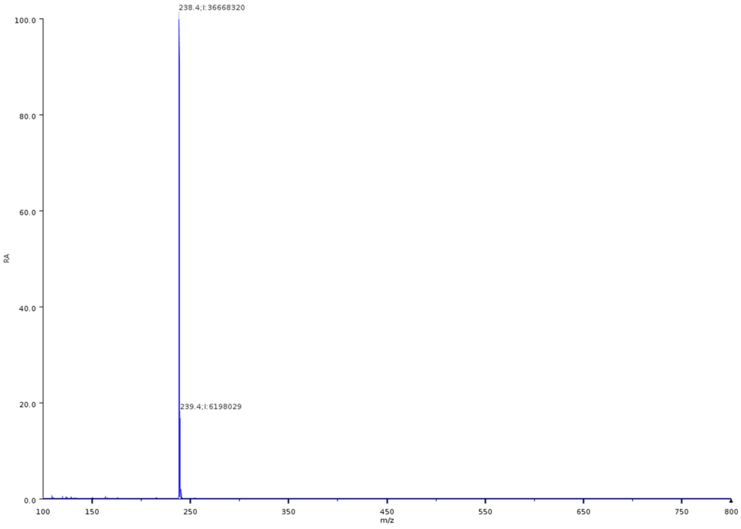

(c)

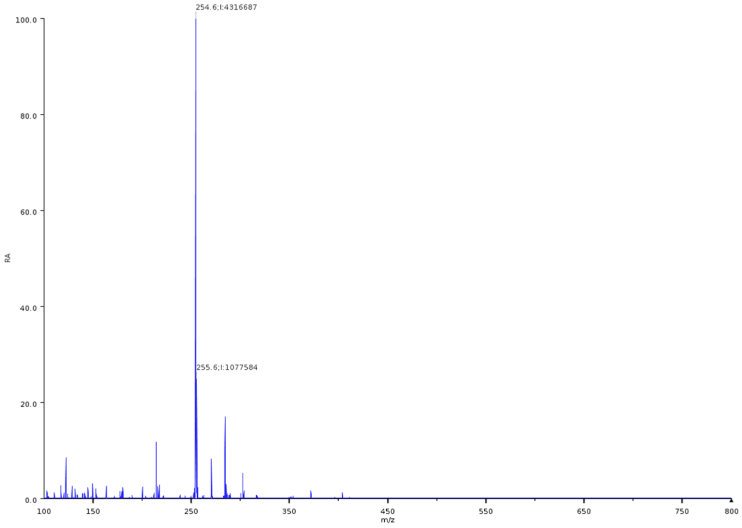

(d)

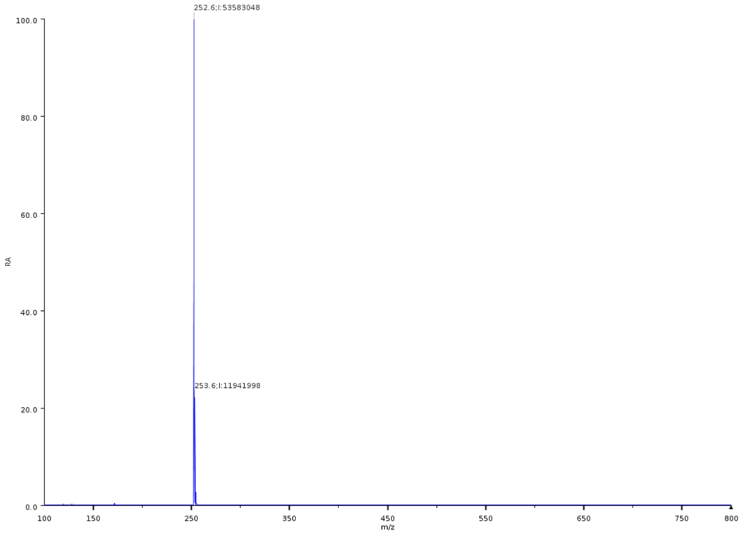

(e)

Figure S4. LC-MS spectra of: (a) **compound 1**; (b) **compound 2**; (c) **compound 3**; (d) **compound 4**; (e) **compound 5**.

1.4. UV-Vis

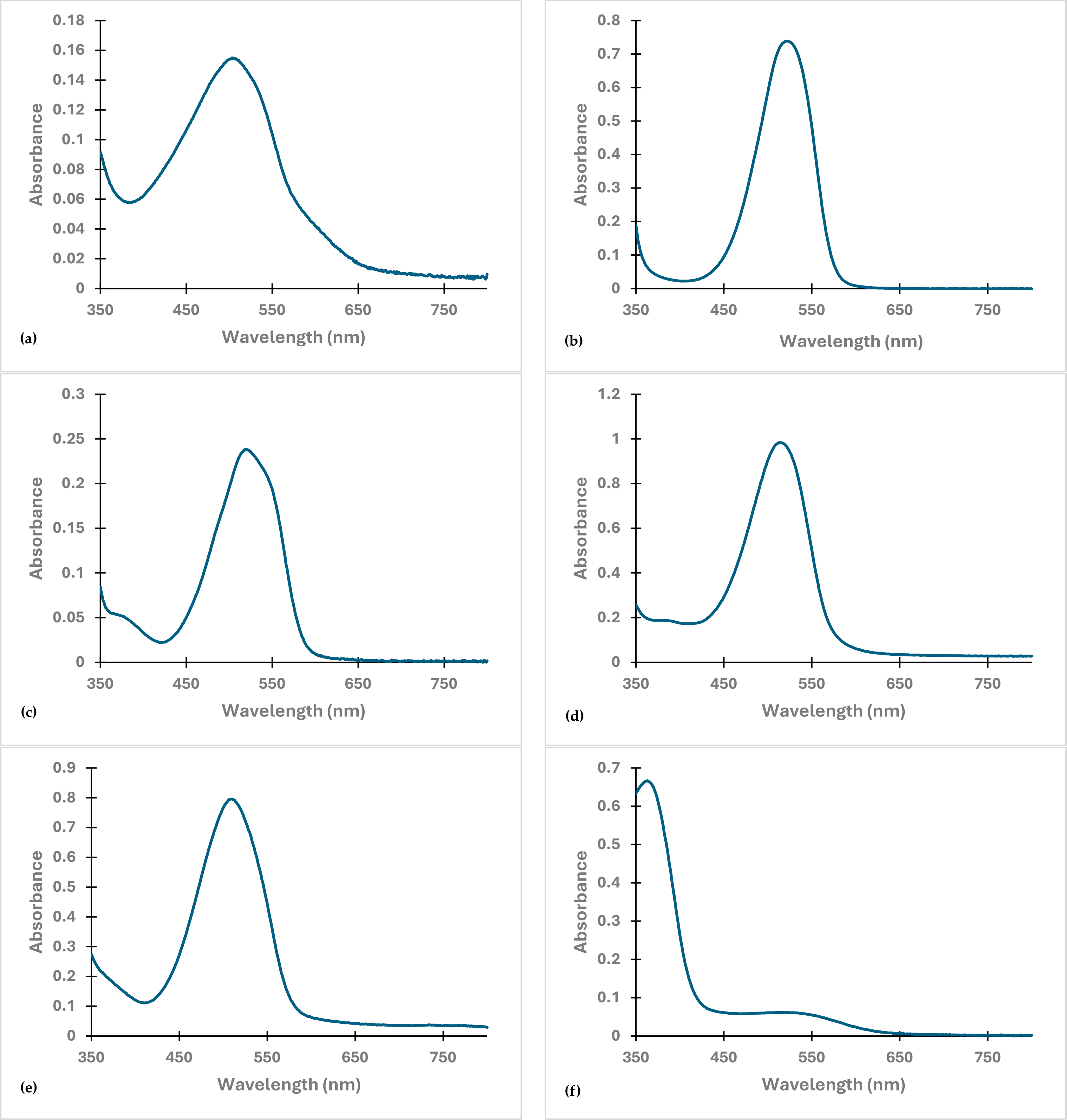

Figure S5. UV-Vis spectrum of: (a) **compound 1**; (b) **compound 2**; (c) **compound 3**; (d) **compound 4**; (e) **compound 5**; (f) **onion peel extract**.

1.5. Thermogravimetric characterization of the dyes and onion peel extract

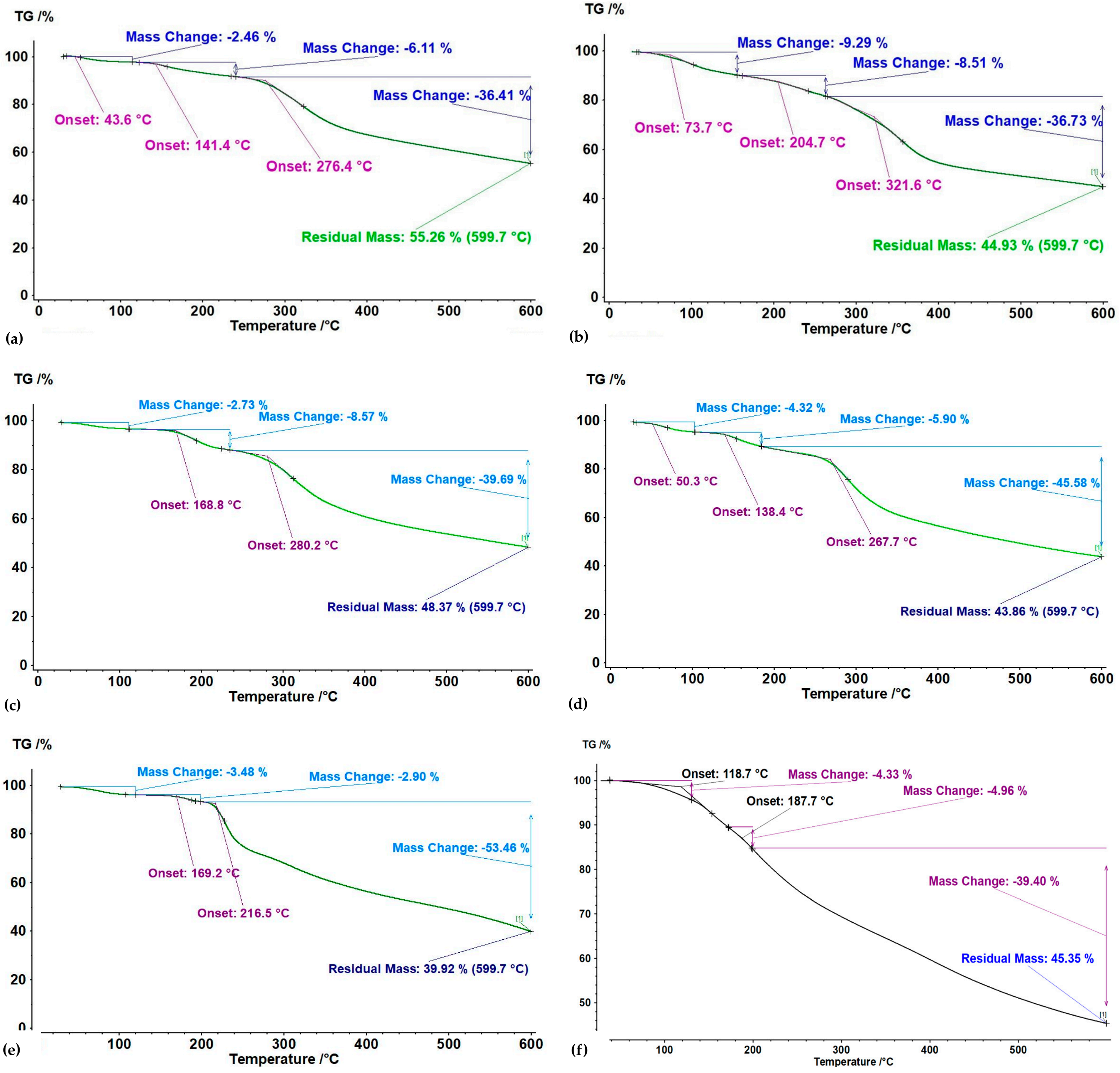

Figure S6. Thermogram of: (a) **compound 1**; (b) **compound 2**; (c) **compound 3**; (d) **compound 4**; (e) **compound 5**; (f) **onion peel extract**.

1.6. pH-dependent photochromic properties

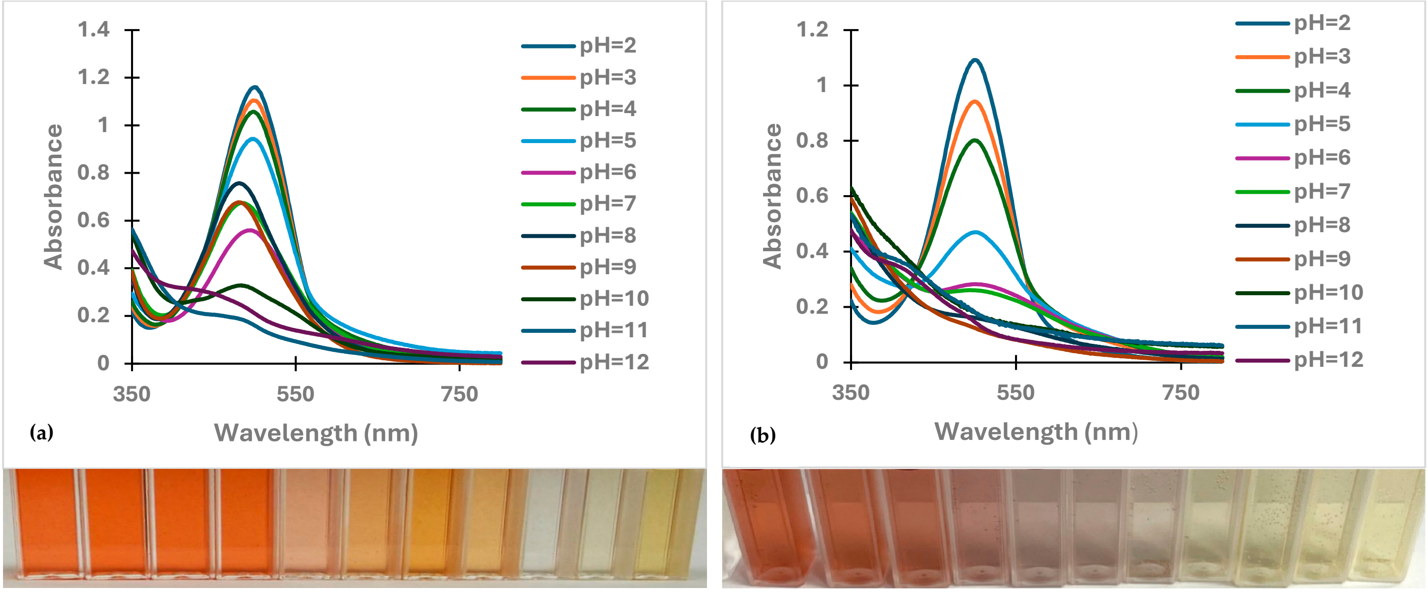

Figure S7. Absorption spectra of the species derived from **compound 1** after a) 0 min and b) 24 h at different pH values.

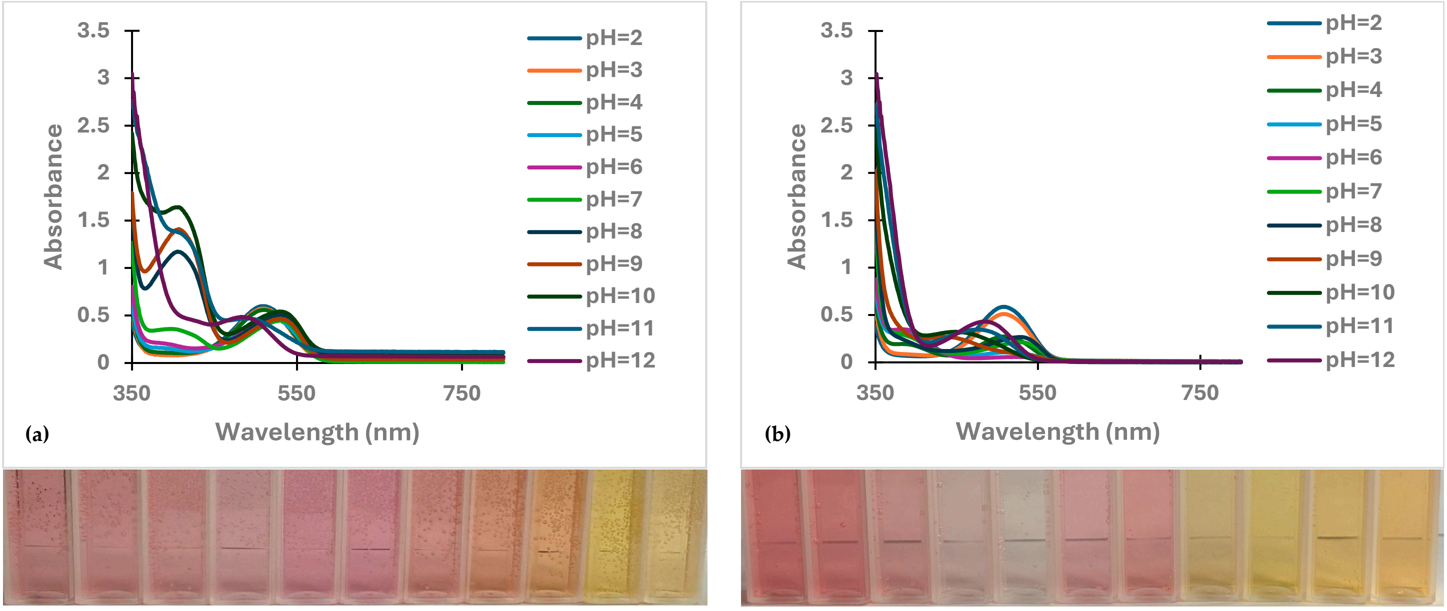

Figure S8. Absorption spectra of the species derived from **compound 2** after a) 0 min and b) 24 h at different pH values.

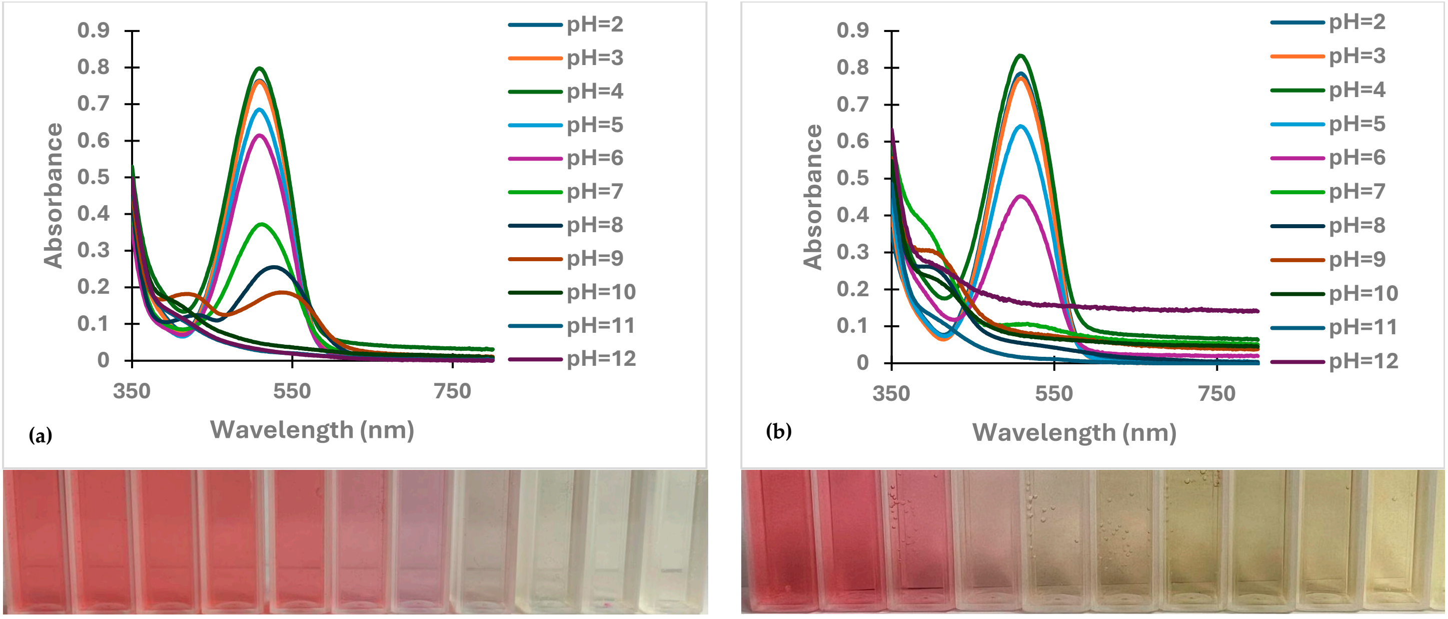

Figure S9. Absorption spectra of the species derived from **compound 3** after a) 0 min and b) 24 h at different pH values.

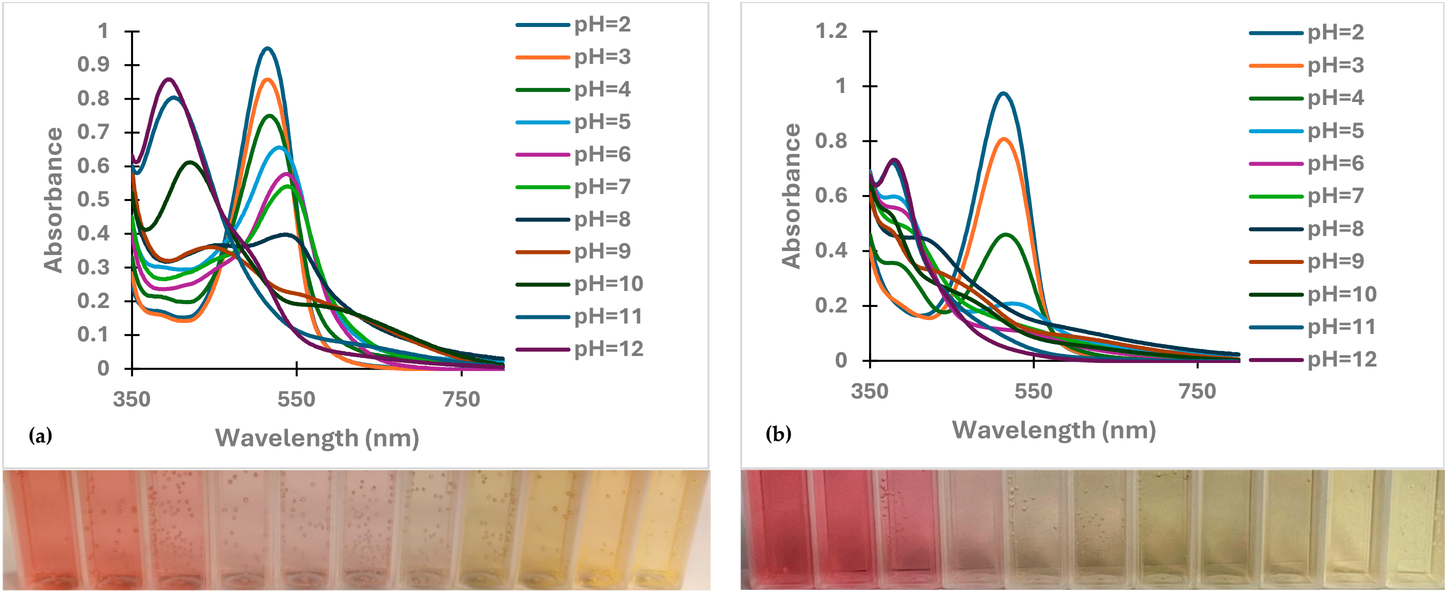

Figure S10. Absorption spectra of the species derived from **compound 4** after a) 0 min and b) 24 h at different pH values.

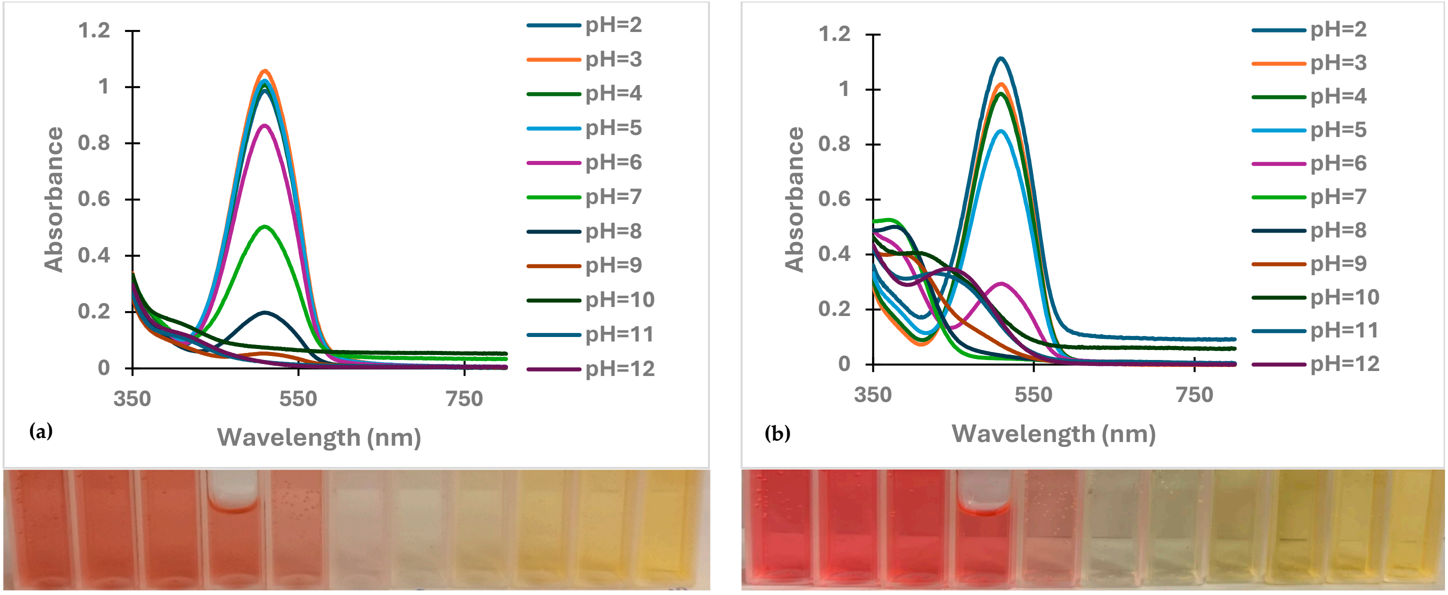

Figure S11. Absorption spectra of the species derived from **compound 5** after a) 0 min and b) 24 h at different pH values.

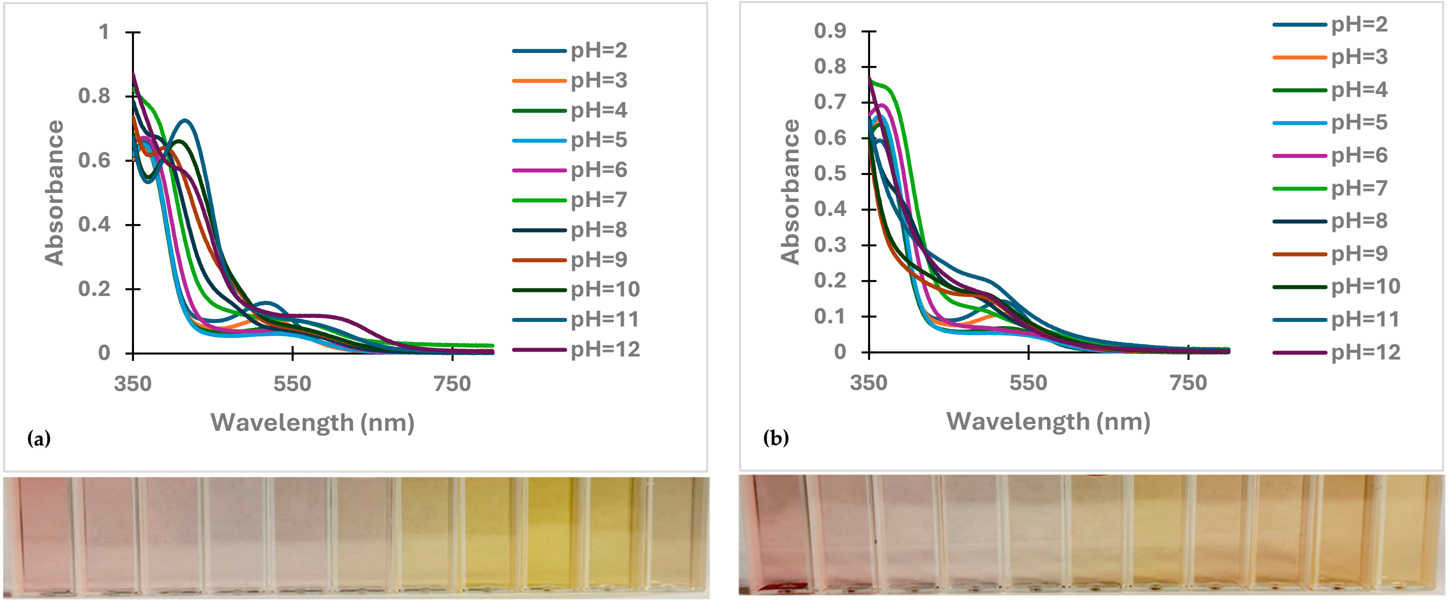

Figure S12. Absorption spectra of the species derived from OPE after a) 0 min and b) 24 h at different pH values.

2. Polymeric films

2.1. FT-IR

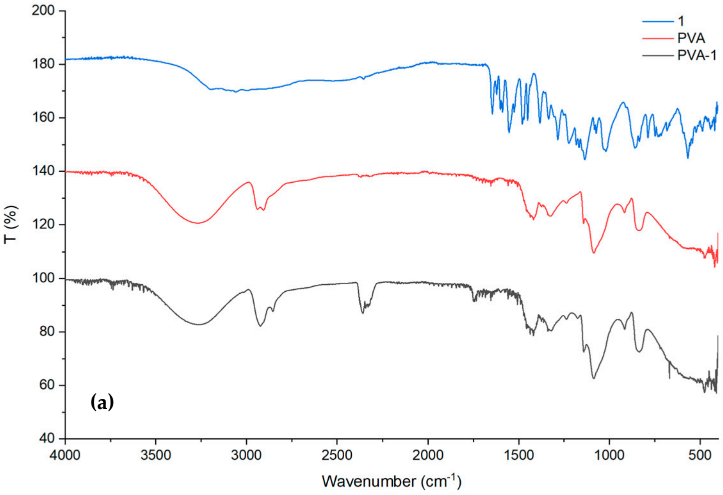

FTIR (ATR) cm^{-1}: 3260 (ν_{OH}), 2923 ($\nu^{as}_{CH_2}$), 2851 ($\nu^{s}_{CH_2}$), 1410 (δ_{CH_2}), 1316 (δ_{OH} + ω_{CH}), 1138 ($\nu_{C\text{-}O}$ crystalline sequence of PVA), 1081 ($\nu_{C\text{-}O}$ + δ_{OH}- amorphous sequence of PVA), 913 (ρ_{CH_2}), 832 (ν_{C-C}).

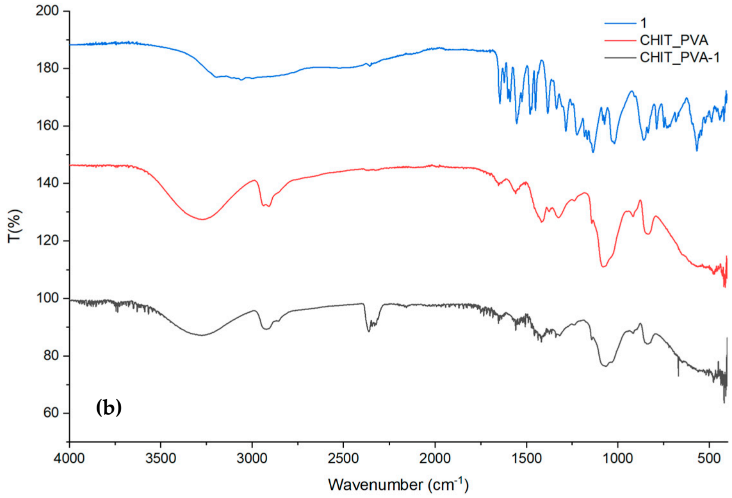

FTIR (ATR) cm^{-1}: 3277 (ν_{OH}), 2923 ($\nu^{as}_{CH_2}$), 2850 ($\nu^{s}_{CH_2}$), 1645 ($\nu_{C=O}$ – amide I-chitosan), 1551 (δ_{NH_2} + $\nu_{C\text{-}N}$), 1415 (δ_{CH_2}), 1319 (δ_{OH} + ω_{CH}), 1145 ($\nu_{C\text{-}O}$ crystalline sequence of PVA), 1077 ($\nu_{C\text{-}O}$ + δ_{OH} - amorphous sequence of PVA + ν^{as}_{COC} - chitosan), 918 (ρ_{CH_2}), 842 (ν_{C-C}).

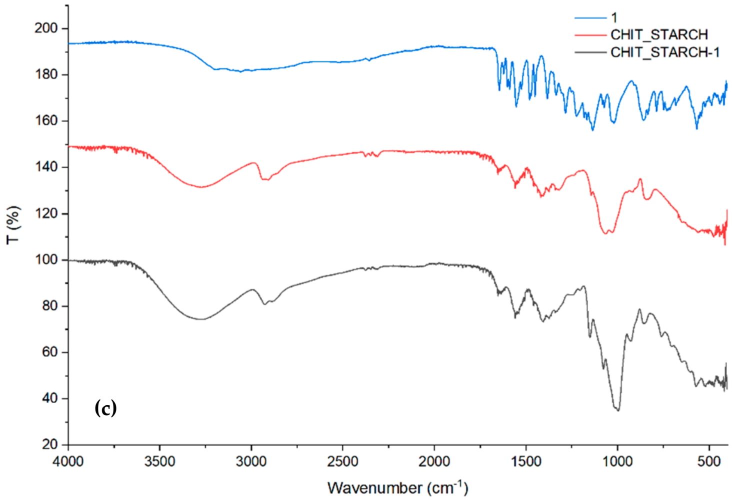

FTIR (ATR) cm^{-1}: 3285 (ν_{OH}), 2924 ($\nu^{as}_{CH_2}$), 1648 ($\nu_{C=O}$ – amide I-chitosan), 1557 (δ_{NH_2} + $\nu_{C\text{-}N}$), 1147 (ν^{s}_{COC}), 1002 (ν^{as}_{COC}).

Figure S13. FTIR spectra of: (a) **dye 1**, PVA (control), and PVA-**1**; (b) **dye 1**, chitosan-PVA (control), and chitosan-PVA-**1**; (c) **dye 1**, chitosan-starch (control), and chitosan-starch-**1**.

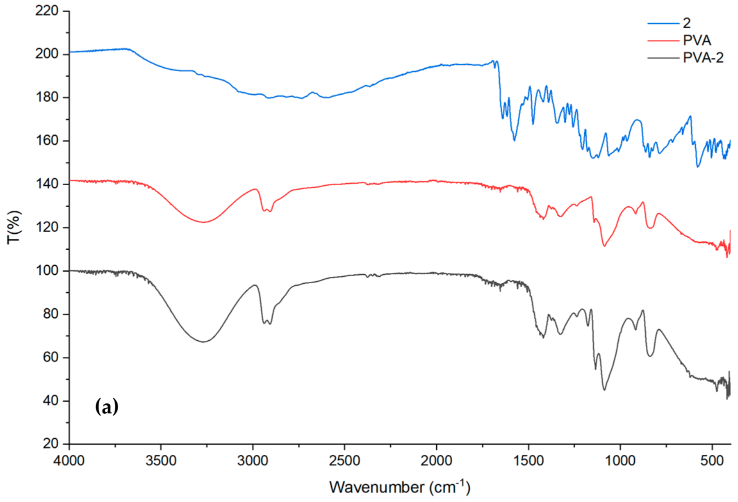

FTIR (ATR) cm^{-1}: 3258 (ν_{OH}), 2940 ($\nu^{as}_{CH_2}$), 2907 ($\nu^{s}_{CH_2}$), 1415 (δ_{CH_2}), 1333 (δ_{OH} + ω_{CH}), 1132 ($\nu_{C\text{-}O}$ crystalline sequence of PVA), 1088 ($\nu_{C\text{-}O}$ + δ_{OH}- amorphous sequence of PVA), 915 (ρ_{CH_2}), 838 (ν_{C-C}).

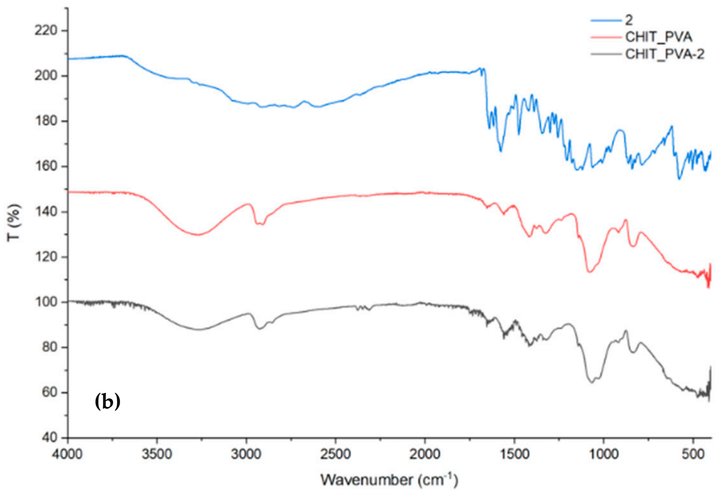

FTIR (ATR) cm^{-1}: 3274 (ν_{OH}), 2927 ($\nu^{as}_{CH_2}$), 2855 ($\nu^{s}_{CH_2}$), 1648 ($\nu_{C=O}$ – amide I-chitosan), 1551 (δ_{NH_2} + $\nu_{C\text{-}N}$), 1411 (δ_{CH_2}), 1316 (δ_{OH} + ω_{CH}), 1145 ($\nu_{C\text{-}O}$ crystalline sequence of PVA), 1023 ($\nu_{C\text{-}O}$ + δ_{OH} - amorphous sequence of PVA + ν^{as}_{COC} - chitosan), 915 (ρ_{CH_2}), 841 (ν_{C-C}).

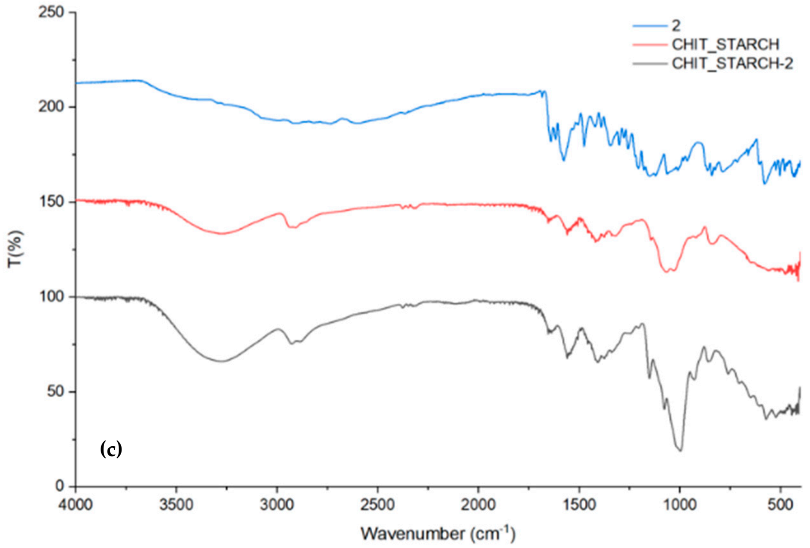

FTIR (ATR) cm^{-1}: 3282 (ν_{OH}), 2929 ($\nu^{as}_{CH_2}$), 1646 ($\nu_{C=O}$ – amide I-chitosan), 1558 (δ_{NH_2} + $\nu_{C\text{-}N}$), 1150 (ν^{s}_{COC}), 1002 (ν^{as}_{COC}).

Figure S14. FTIR spectra of: (a) **dye 2**, PVA (control), and PVA-**2**; (b) **dye 2**, chitosan-PVA (control), and chitosan-PVA-**2**; (c) **dye 2**, chitosan-starch (control), and chitosan-starch-**2**.

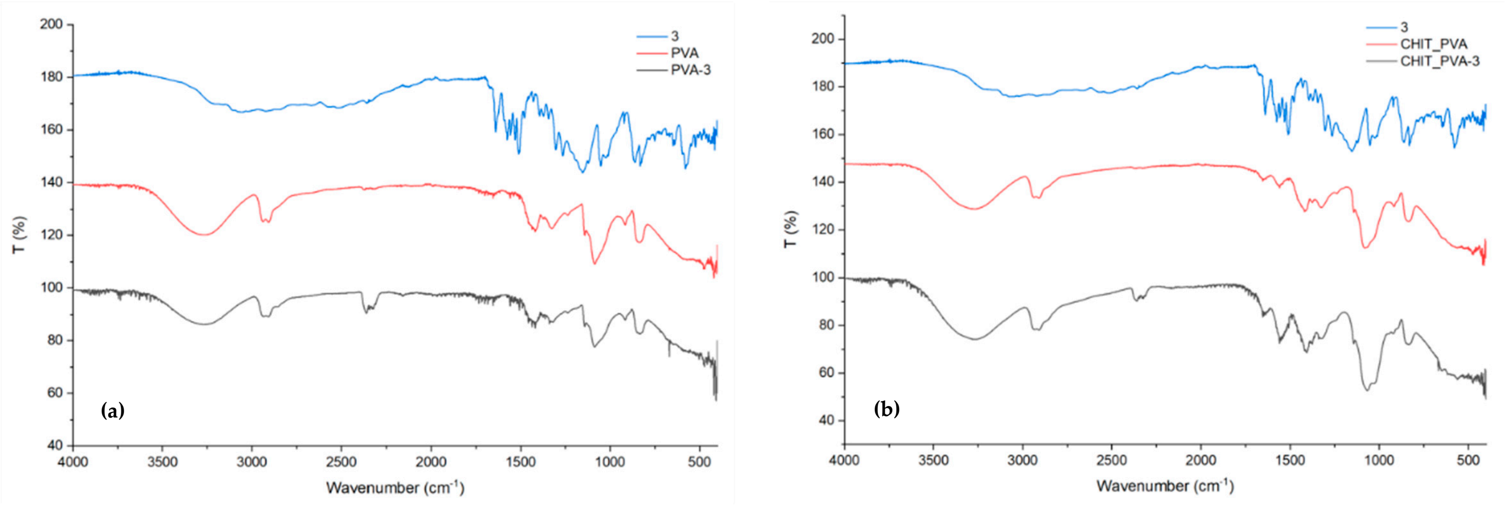

FTIR (ATR) cm^{-1}: 3266 (ν_{OH}), 2937 ($\nu^{as}_{CH_2}$), 2909 ($\nu^{s}_{CH_2}$), 1416 (δ_{CH_2}), 1319 (δ_{OH} + ω_{CH}), 1140 ($\nu_{C\text{-}O}$ crystalline sequence of PVA), 1091 ($\nu_{C\text{-}O}$ + δ_{OH}- amorphous sequence of PVA), 916 (ρ_{CH_2}), 834 (ν_{C-C}).

FTIR (ATR) cm^{-1}: 3278 (ν_{OH}), 2941 ($\nu^{as}_{CH_2}$), 2901 ($\nu^{s}_{CH_2}$), 1634 ($\nu_{C=O}$– amide I-chitosan), 1557 (δ_{NH_2} + $\nu_{C\text{-}N}$), 1412 cm^{-1} (δ_{CH_2}), 1372 (δ_{OH} + ω_{CH}), 1143 ($\nu_{C\text{-}O}$ crystalline sequence of PVA), 1024 ($\nu_{C\text{-}O}$ + δ_{OH} - amorphous sequence of PVA + ν^{as}_{COC} - chitosan), 915 (ρ_{CH_2}), 837 (ν_{C-C}).

Figure S15. FTIR spectra of: (a) **dye 3**, PVA (control), and PVA-**3**; (b) **dye 3**, chitosan-PVA (control), and chitosan-PVA-**3**.

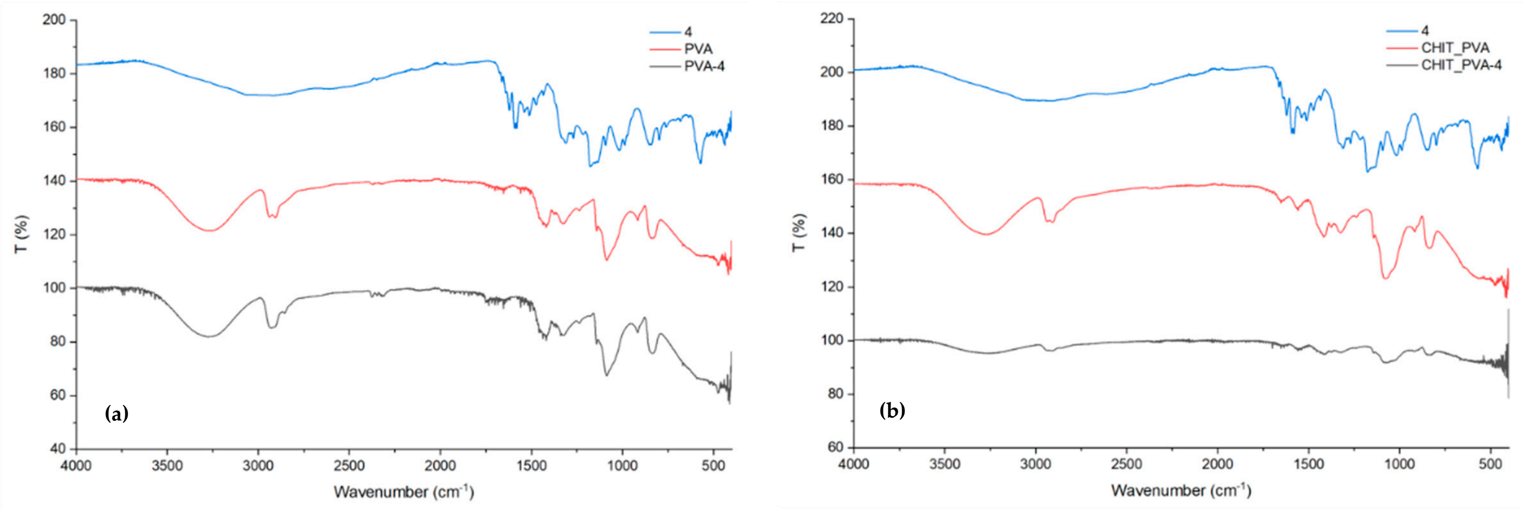

FTIR (ATR) cm^{-1}: 3270 (ν_{OH}), 2912 ($\nu^{as}_{CH_2}$), 2856 ($\nu^{s}_{CH_2}$), 1420 (δ_{CH_2}), 1329 (δ_{OH} + ω_{CH}), 1139 ($\nu_{C\text{-}O}$ crystalline sequence of PVA), 1085 ($\nu_{C\text{-}O}$ + δ_{OH}- amorphous sequence of PVA), 915 (ρ_{CH_2}), 830 (ν_{C-C}).

FTIR (ATR) cm^{-1}: 3298 (ν_{OH}), 2937 ($\nu^{as}_{CH_2}$), 2901 ($\nu^{s}_{CH_2}$), 1643 ($\nu_{C=O}$– amide I-chitosan), 1556 (δ_{NH_2} + $\nu_{C\text{-}N}$), 1409 (δ_{CH_2}), 1326 (δ_{OH} + ω_{CH}), 1143 ($\nu_{C\text{-}O}$ crystalline sequence of PVA), 1078 ($\nu_{C\text{-}O}$ + δ_{OH} - amorphous sequence of PVA + ν^{as}_{COC} - chitosan), 921 (ρ_{CH_2}), 858 (ν_{C-C}).

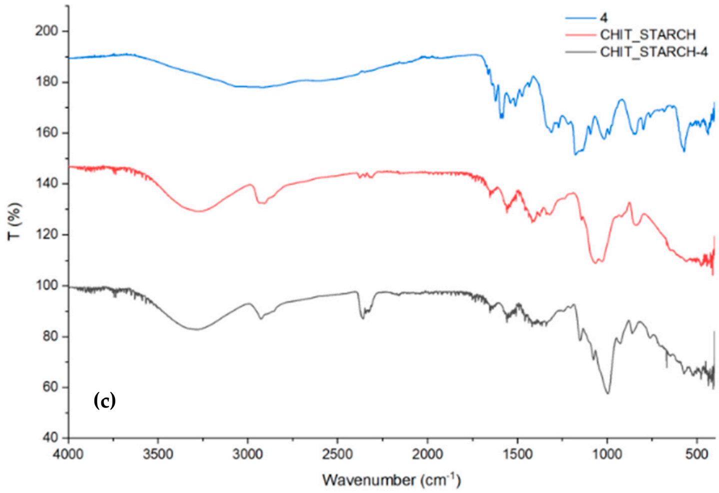

FTIR (ATR) cm^{-1}: 3273 (ν_{OH}), 2925 ($\nu^{as}_{CH_2}$), 1648 ($\nu_{C=O}$ – amide I-chitosan), 1561 (δ_{NH_2} + $\nu_{C\text{-}N}$), 1152 (ν^{s}_{COC}), 1078 (ν^{as}_{COC}).

Figure S16. FTIR spectra of: (a) **dye 4**, PVA (control), and PVA-**4**; (b) **dye 4**, chitosan-PVA (control), and chitosan-PVA-**4**; (c) **dye 4**, chitosan-starch (control), and chitosan-starch-**4**.

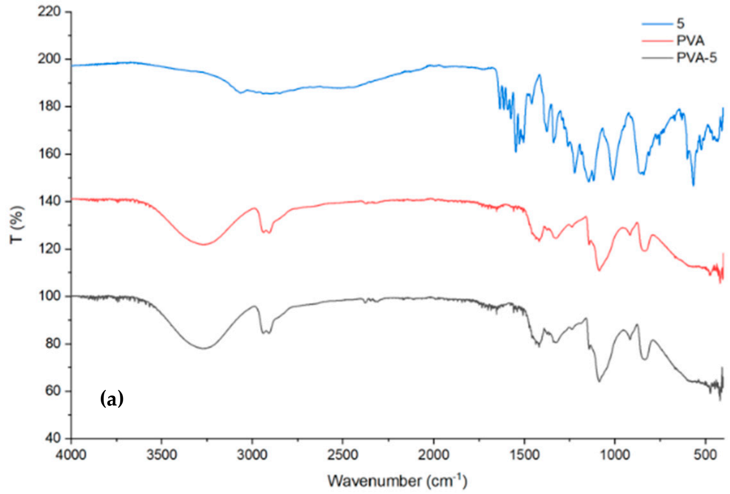

FTIR (ATR) cm^{-1}: 3268 (ν_{OH}), 2936 ($\nu^{as}_{CH_2}$), 2909 ($\nu^{s}_{CH_2}$), 1420 (δ_{CH_2}), 1322 (δ_{OH} + ω_{CH}), 1142 ($\nu_{C\text{-}O}$ crystalline sequence of PVA), 1090 ($\nu_{C\text{-}O}$ + δ_{OH}- amorphous sequence of PVA), 913 (ρ_{CH_2}), 832 (ν_{C-C}).

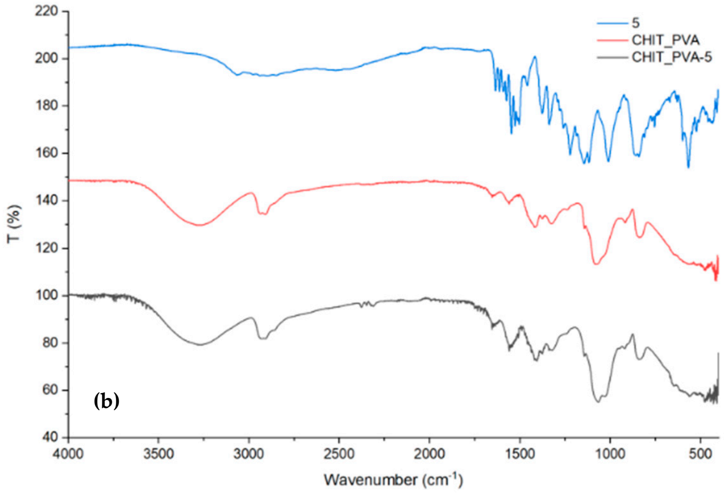

FTIR (ATR) cm^{-1}: 3278 (ν_{OH}), 2927 ($\nu^{as}_{CH_2}$), 2856 ($\nu^{s}_{CH_2}$), 1648 ($\nu_{C=O}$ – amide I-chitosan), 1560 (δ_{NH_2} + $\nu_{C\text{-}N}$), 1408 (δ_{CH_2}), 1340 (δ_{OH} + ω_{CH}), 1150 ($\nu_{C\text{-}O}$ crystalline sequence of PVA), 1053 ($\nu_{C\text{-}O}$ + δ_{OH} - amorphous sequence of PVA + ν^{as}_{COC} - chitosan), 930 (ρ_{CH_2}), 849 (ν_{C-C}).

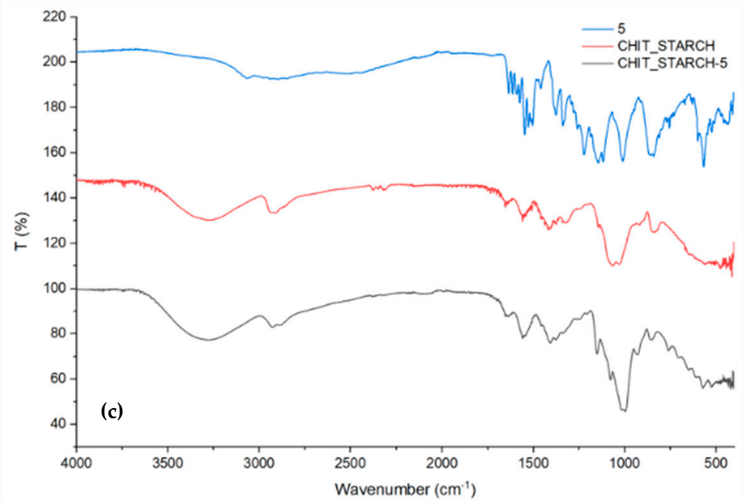

FTIR (ATR) cm^{-1}: 3282 (ν_{OH}), 2925 ($\nu^{as}_{CH_2}$), 1646 ($\nu_{C=O}$ – amide I-chitosan), 1560 (δ_{NH_2} + ν_{C-N}), 1150 (ν^{s}_{COC}), 1076 (ν^{as}_{COC}).

Figure S17. FTIR spectra of: (a) **dye 5**, PVA (control), and PVA-**5**; (b) **dye 5**, chitosan-PVA (control), and chitosan-PVA-**5**; (c) **dye 5**, chitosan-starch (control), and chitosan-starch-**5**.

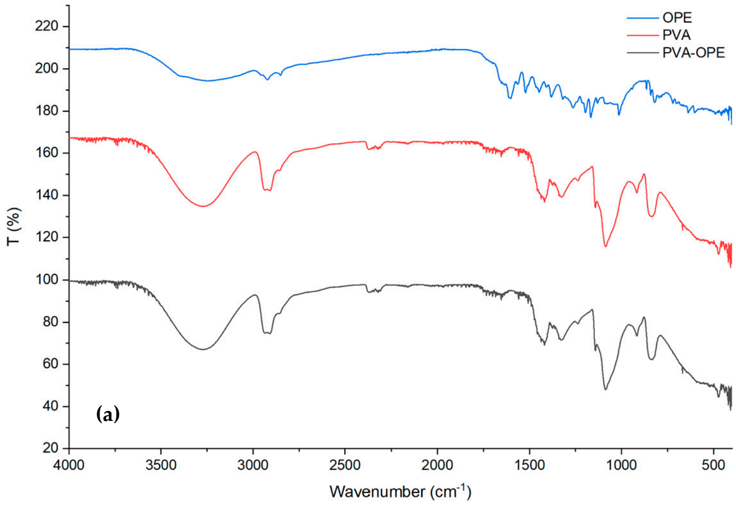

FTIR (ATR) cm^{-1}: 3273 (ν_{OH}), 2935 ($\nu^{as}_{CH_2}$), 2902 ($\nu^{s}_{CH_2}$), 1424 (δ_{CH_2}), 1322 (δ_{OH} + ω_{CH}), 1139 (ν_{C-O} crystalline sequence of PVA), 1090 (ν_{C-O} + δ_{OH}- amorphous sequence of PVA), 919 (ρ_{CH_2}), 837 (ν_{C-C}).

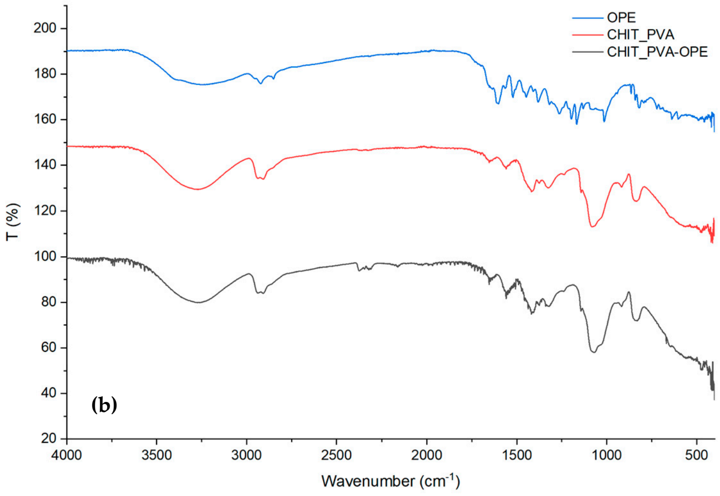

FTIR (ATR) cm^{-1}: 3273 (ν_{OH}), 2937 ($\nu^{as}_{CH_2}$), 2906 ($\nu^{s}_{CH_2}$), 1646 ($\nu_{C=O}$ – amide I-chitosan), 1557 (δ_{NH_2} + ν_{C-N}), 1416 cm^{-1} (δ_{CH_2}), 1327 (δ_{OH} + ω_{CH}), 1144 (ν_{C-O} crystalline sequence of PVA), 1074 (ν_{C-O} + δ_{OH} - amorphous sequence of PVA + ν^{as}_{COC} - chitosan), 915 (ρ_{CH_2}), 836 (ν_{C-C}).

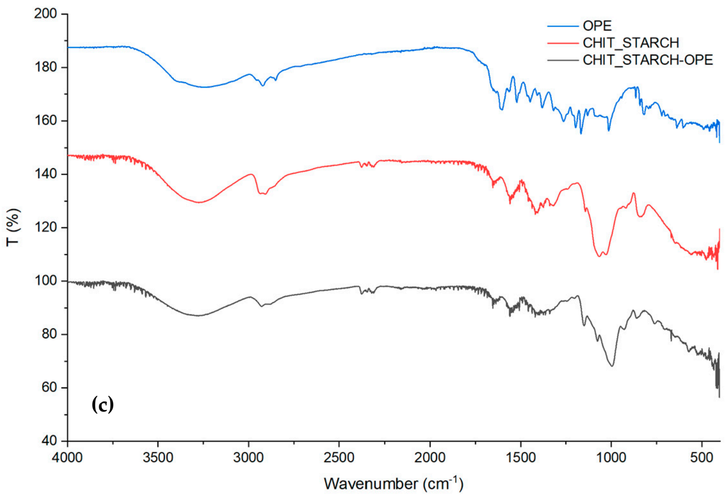

FTIR (ATR) cm^{-1}: 3275 (ν_{OH}), 2924 ($\nu_{CH_2}^{as}$), 1655 ($\nu_{C=O}$ – amide I-chitosan), 1556 (δ_{NH_2} + $\nu_{C\text{-}N}$), 1152 (ν_{COC}^{s}), 1078 (ν_{COC}^{s}), 999 (ν_{COC}^{as}), 842 (δ_{CH} pyranose ring).

Figure S18. FTIR spectra of: (a) **OPE**, PVA (control), and PVA-**OPE**; (b) **OPE**, chitosan-PVA (control), and chitosan-PVA-**OPE**; (c) **OPE**, chitosan-starch (control), and chitosan-starch-**OPE**.

2.2. UV-Vis spectroscopy

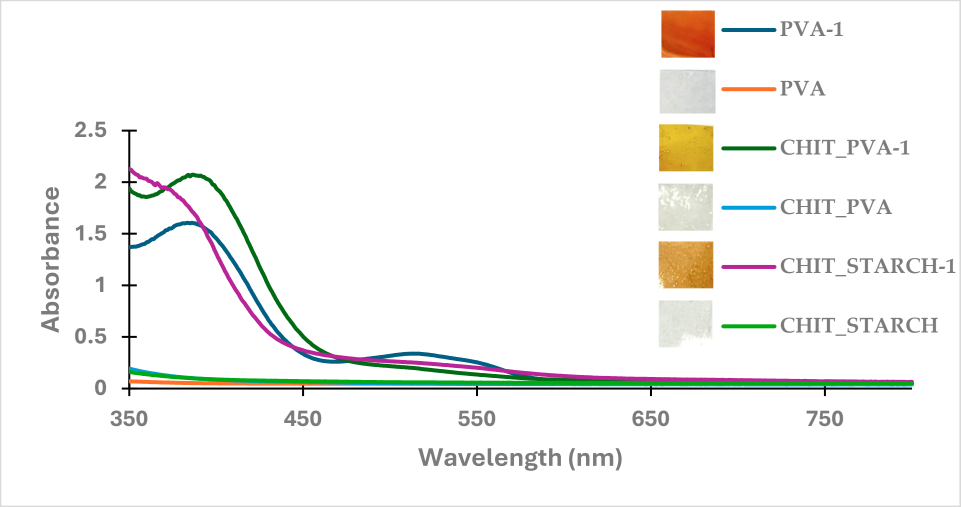

Figure S19. UV-Vis spectra of bio-based polymeric films (control and with **compound 1**).

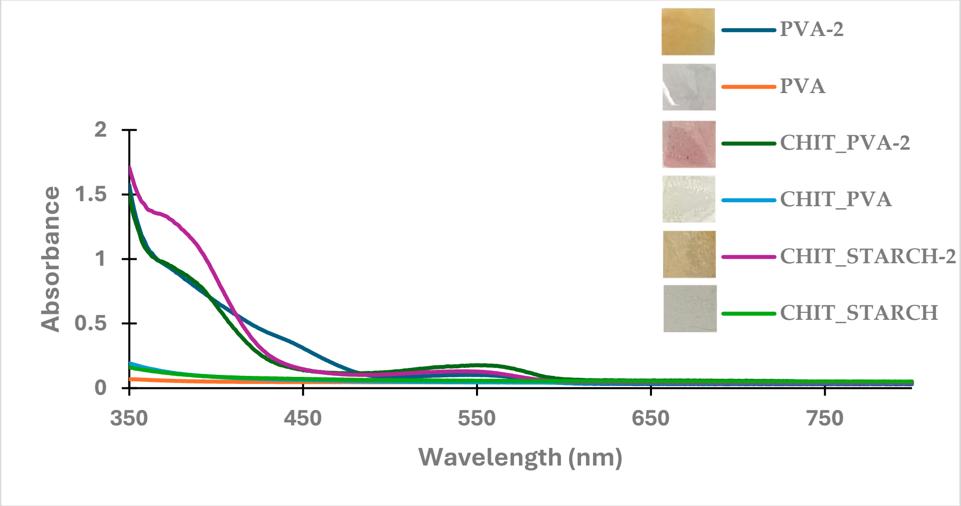

Figure S20. UV-Vis spectra of bio-based polymeric films (control and with **compound 2**).

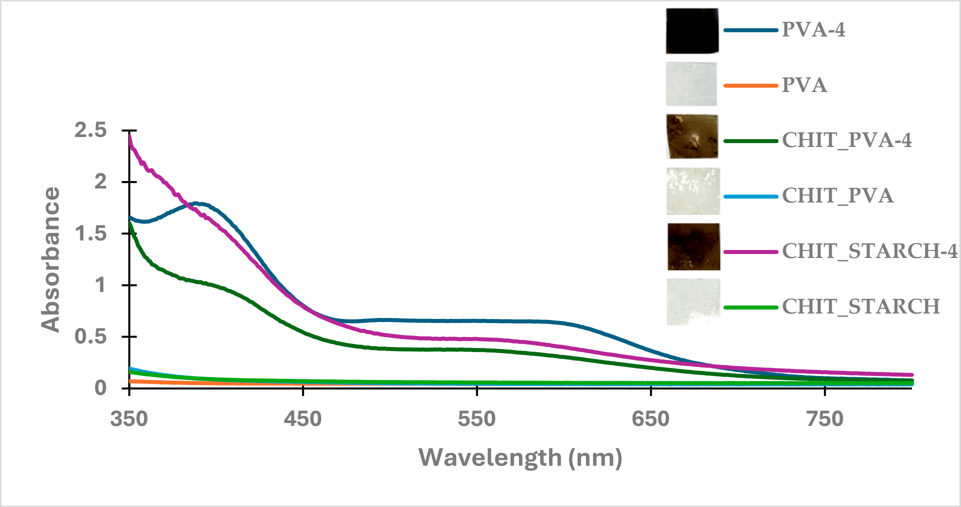

Figure S21. UV-Vis spectra of bio-based polymeric films (control and with **compound 4**).

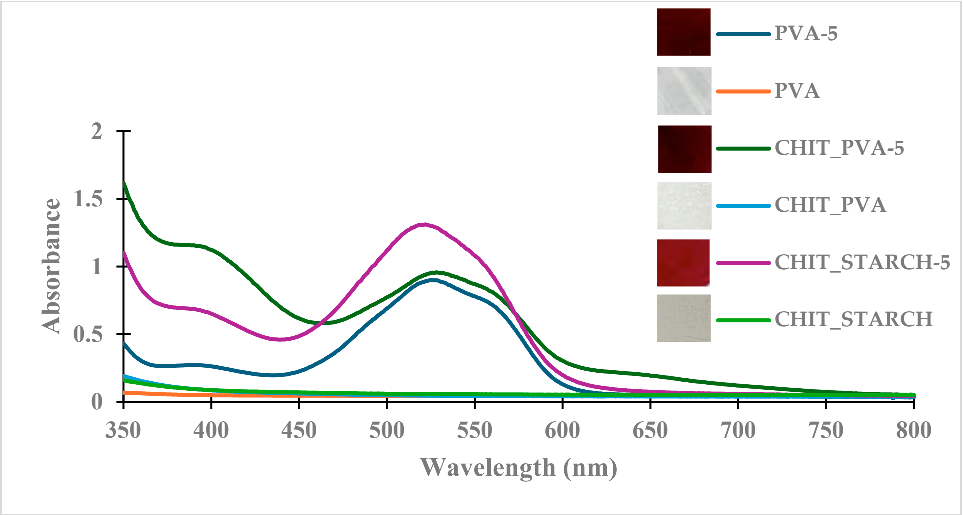

Figure S22. UV-Vis spectra of bio-based polymeric films (control and with **compound 5**).

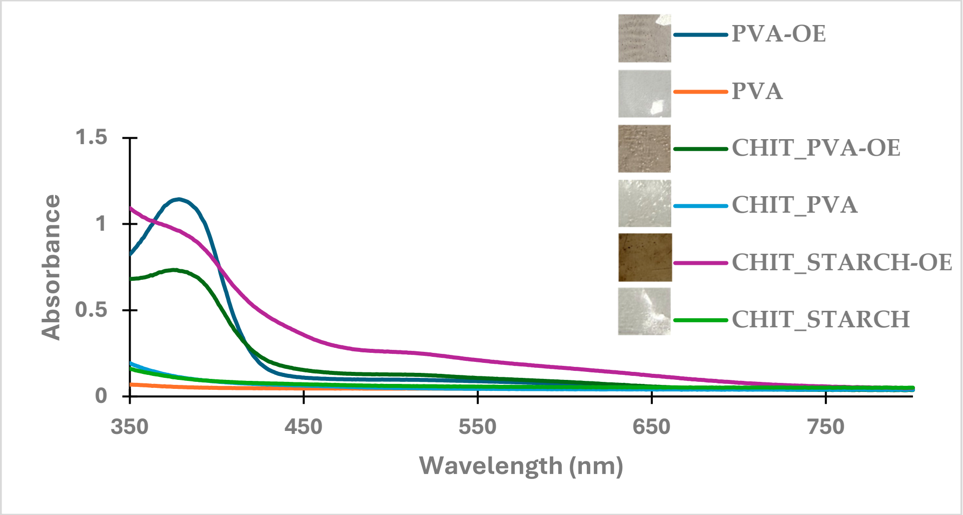

Figure S23. UV-Vis spectra of bio-based polymeric films (control and with **onion peel extract**).

2.3. *Thermal Analysis*

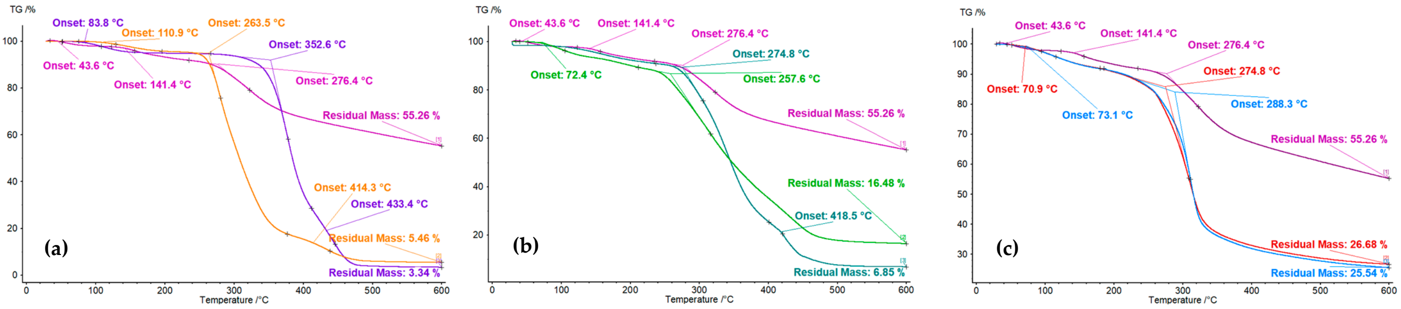

Figure S24. Thermograms of: (**a**) PVA–**1** film (violet), PVA film (orange), and **compound 1** (fuchsia); (**b**) chitosan–PVA–**1** film (light green), chitosan–PVA film (dark green), and **compound 1** (fuchsia); (**c**) chitosan–starch–**1** film (blue), chitosan-starch film (red), and **compound 1** (fuchsia).

Table S1. Weight losses of the blank and **compound 1** incorporated PVA, chitosan-PVA, chitosan-starch films and **compound 1.**

Compound	Weight loss [%]				
	25-200°C	25-300°C	25-400°C	25-500°C	25-600°C
1	6.73	15.51	32.69	39.07	44.74
PVA	4.30	43.23	84.65	94.07	94.53
PVA_1	5.09	6.26	64.79	96.17	96.65
CHIT_PVA	7.63	22.12	74.45	92.39	93.14
CHIT_PVA-1	9.88	31.43	64.72	82.22	83.52
CHIT_STARCH	9.16	37.34	67.03	71.30	73.32
CHIT_STARCH-1	9.08	33.36	68.08	72.22	74.45

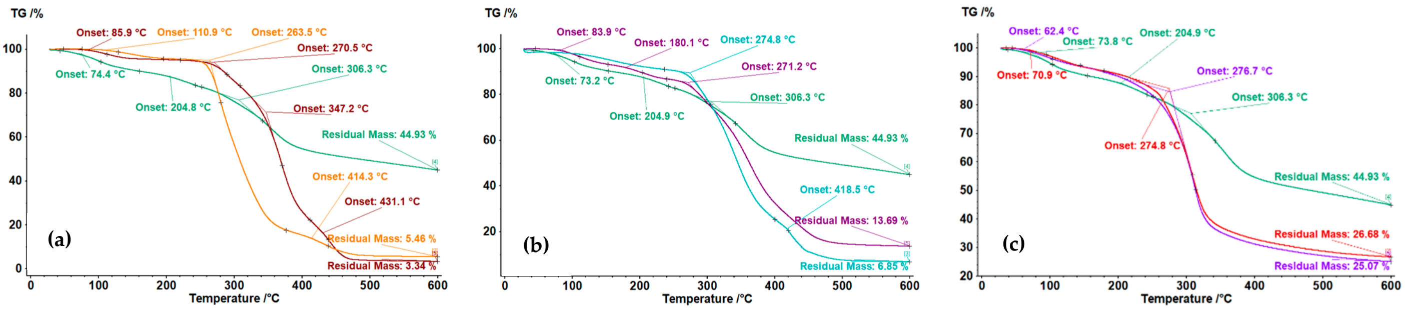

Figure S25. Thermograms of: (**a**) PVA–**2** film (brown), PVA film (orange), and **compound 2** (green); **b**) chitosan–PVA–**2** film (purple), chitosan–PVA film (azure), and **compound 2** (green); **c**) chitosan–starch–**2** film (purple), chitosan-starch film (red), and **compound 2** (green).

Table S2. Weight losses of the blank and **compound 2** incorporated PVA, chitosan-PVA, chitosan-starch films and **compound 2**.

Compound	Weight loss [%]				
	25-200°C	25-300°C	25-400°C	25-500°C	25-600°C
2	11.90	23.51	45.05	50.46	54.75
PVA	4.30	43.23	84.65	94.07	94.53
PVA_2	4.82	14.50	73.80	96.21	96.65
CHIT_PVA	7.63	22.12	74.45	92.39	93.14
CHIT_PVA-2	10.11	23.57	67.41	85.24	86.30
CHIT_STARCH	9.16	37.34	67.03	71.30	73.32
CHIT_STARCH-2	9.94	37.60	68.76	72.84	74.91

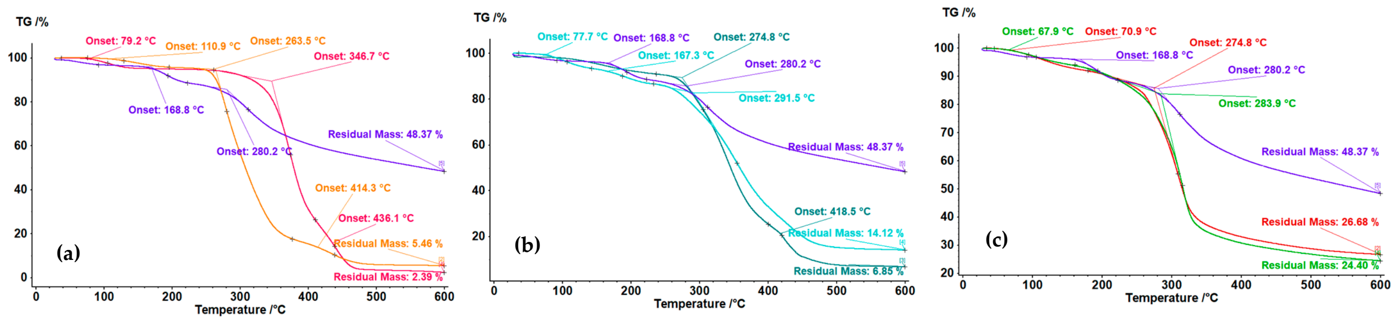

Figure S26. Thermograms of: (**a**) PVA–**3** film (pink), PVA film (orange), and **compound 3** (purple); (**b**) chitosan–PVA–**3** film (azure), chitosan–PVA film (dark green), and **compound 3** (purple); **c**) chitosan–starch–**3** film (green), chitosan-starch film (red), and **compound 3** (purple).

Table S3. Weight losses of the blank and **compound 3** incorporated PVA, chitosan-PVA, chitosan-starch films and **compound 3**.

Compound	Weight loss [%]				
	25-200°C	25-300°C	25-400°C	25-500°C	25-600°C
3	9.04	20.06	39.16	46.21	51.61
PVA	4.30	43.23	84.65	94.07	94.53
PVA-3	5.06	7.84	68.71	96.53	97.60
CHIT_PVA	7.63	22.12	74.45	92.39	93.14
CHIT_PVA-3	11.15	25.35	66.86	84.75	85.87
CHIT_STARCH	9.16	37.34	67.03	71.30	73.32
CHIT_STARCH-3	8.81	35.00	69.26	73.25	75.59

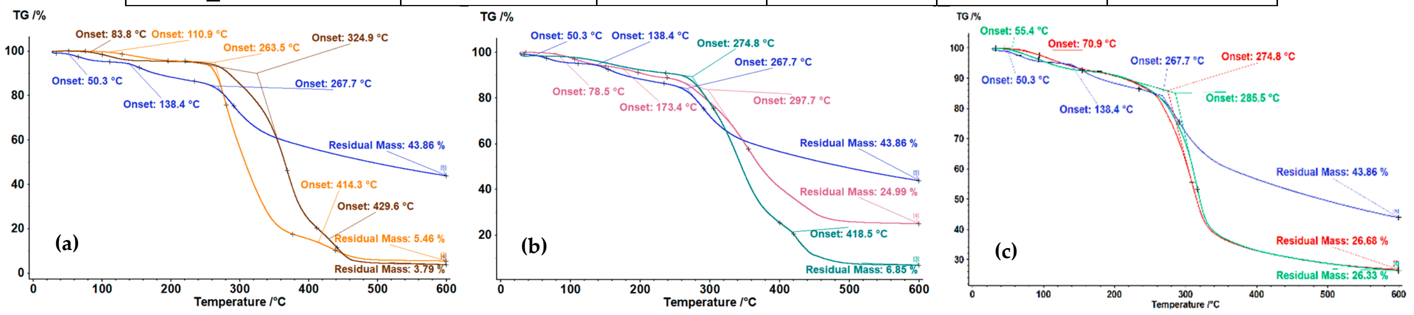

Figure S27. Thermograms of: (**a**) PVA–**4** film (brown), PVA film (orange), and **compound 4** (purple); (**b**) chitosan–PVA–**4** film (pink), chitosan–PVA film (dark green), and **compound 4** (purple); (**c**) chitosan–starch–**4** film (green), chitosan-starch film (red), and **compound 4** (purple).

Table S4. Weight losses of the blank and **compound 4** incorporated PVA, chitosan-PVA, chitosan-starch films and **compound 4**.

Compound	Weight loss [%]				
	25-200°C	25-300°C	25-400°C	25-500°C	25-600°C
4	11.17	27.31	42.91	50.08	55.61
PVA	4.30	43.23	84.65	94.07	94.53
PVA-4	4.64	14.63	75.70	95.48	96.19
CHIT_PVA	7.63	22.12	74.45	92.39	93.14
CHIT_PVA-4	9.05	21.83	59.07	74.08	75.00
CHIT_STARCH	9.16	37.34	67.03	71.30	73.32
CHIT_STARCH-4	8.98	31.63	66.99	71.30	73.67

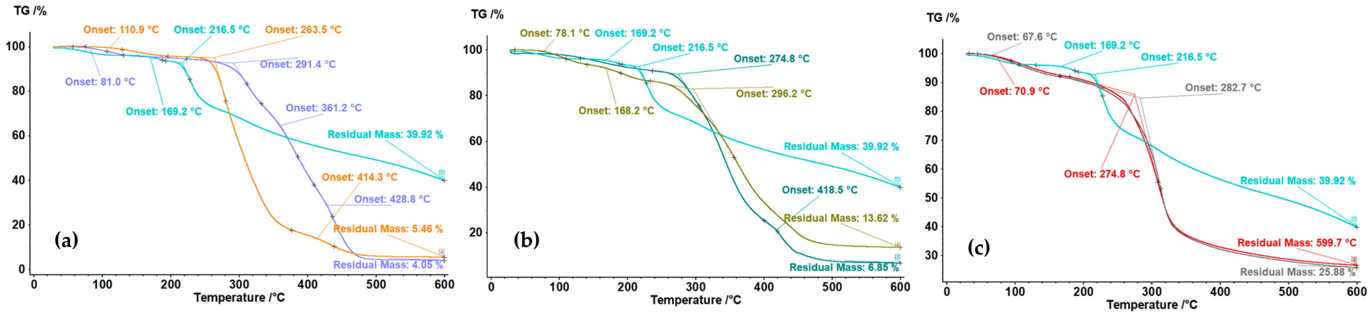

Figure S28. Thermograms of: (**a**) PVA–**5** film (purple), PVA film (orange), and **compound 5** (azure); (**b**) chitosan–PVA–**5** film (olive), chitosan–PVA film (dark green), and **compound 5** (azure); (**c**) chitosan–starch–**5** film (grey), chitosan-starch film (red), and **compound 5** (azure).

Table S5. Weight losses of the blank and **compound 5** incorporated PVA, chitosan-PVA, chitosan-starch films and **compound 5**.

Compound	Weight loss [%]				
	25-200°C	25-300°C	25-400°C	25-500°C	25-600°C
5	6.20	31.50	43.15	50.43	59.62
PVA	4.30	43.23	84.65	94.07	94.53
PVA-5	5.03	12.13	57.29	95.60	95.93
CHIT_PVA	7.63	22.12	74.45	92.39	93.14
CHIT_PVA-5	11.24	24.27	66.59	85.25	86.38
CHIT_STARCH	9.16	37.34	67.03	71.30	73.32
CHIT_STARCH-5	9.74	34.53	67.78	71.96	74.12

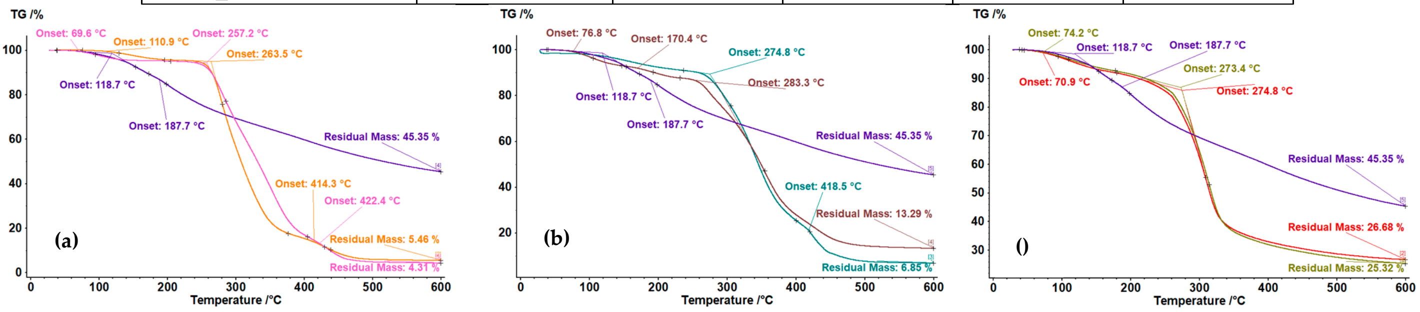

Figure S29. Thermograms of: (**a**) PVA–**OPE** film (pink), PVA film (orange), and **OPE** (purple); (**b**) chitosan–PVA–**OPE** film (brown), chitosan–PVA film (dark green), and **OPE** (purple); (**c**) chitosan–starch–**OPE** film (olive), chitosan-starch film (red), and **OPE** (purple).

Table S6. Weight losses of the blank and **OPE** incorporated PVA, chitosan-PVA, chitosan-starch films and **OPE.**

Compound	Weight loss [%]				
	25-200°C	25-300°C	25-400°C	25-500°C	25-600°C
OPE	15.50	30.62	40.62	48.92	54.64
PVA	4.30	43.23	84.65	94.07	94.53
PVA-OPE	4.86	31.83	83.00	95.21	95.68
CHIT_PVA	7.63	22.12	74.45	92.39	93.14
CHIT_PVA-OPE	10.47	27.33	72.00	85.63	86.70
CHIT_STARCH	9.16	37.34	67.03	71.30	73.32
CHIT_STARCH-OPE	8.49	35.18	68.04	72.52	74.68

2.4. *Films' sensitivity to pH changes*

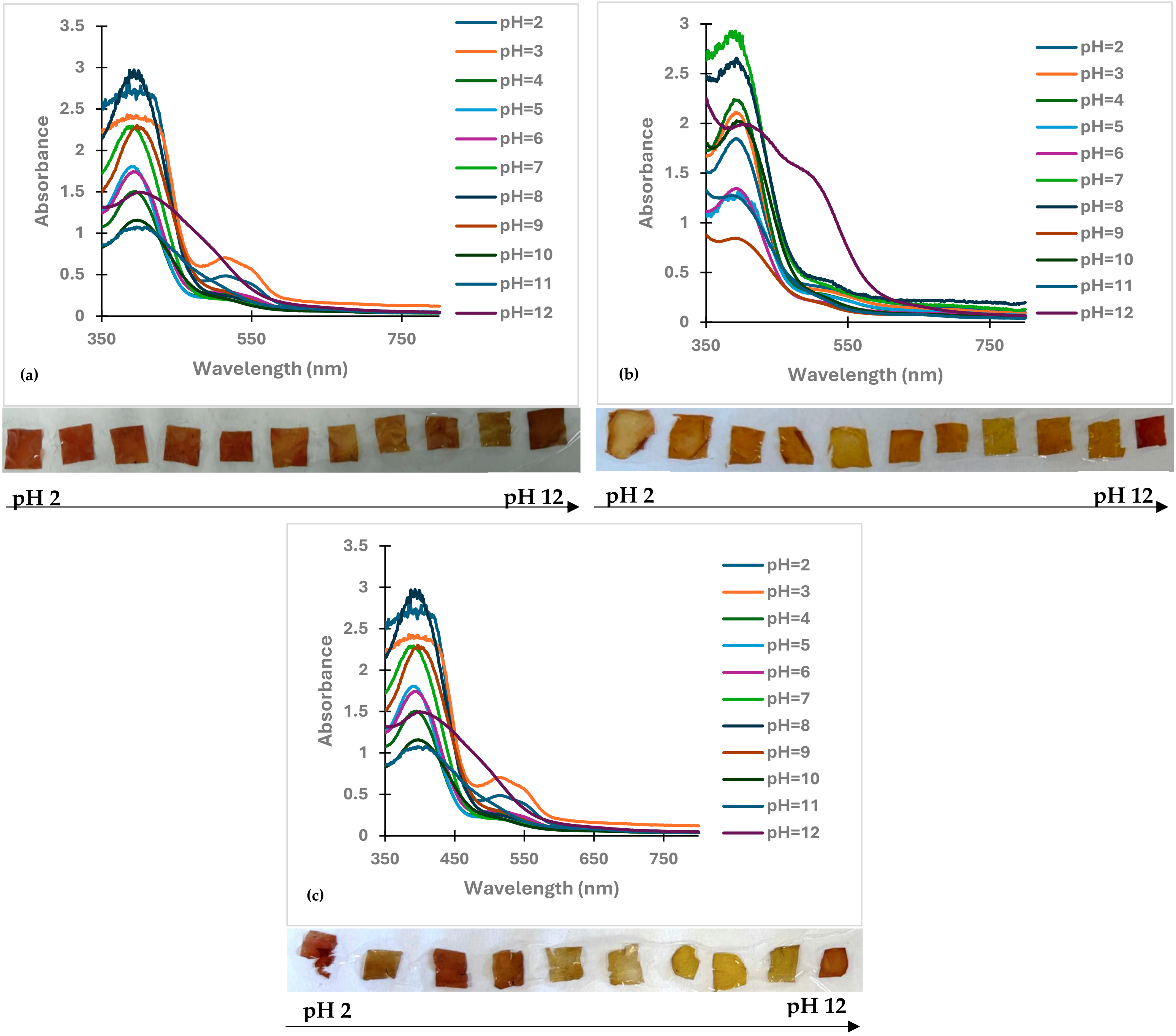

Figure S30. UV-Vis spectra of: (a) PVA-**1** film; (b) chitosan-PVA-**1** film; (c) chitosan-starch-**1** film after 2h in buffer solutions.

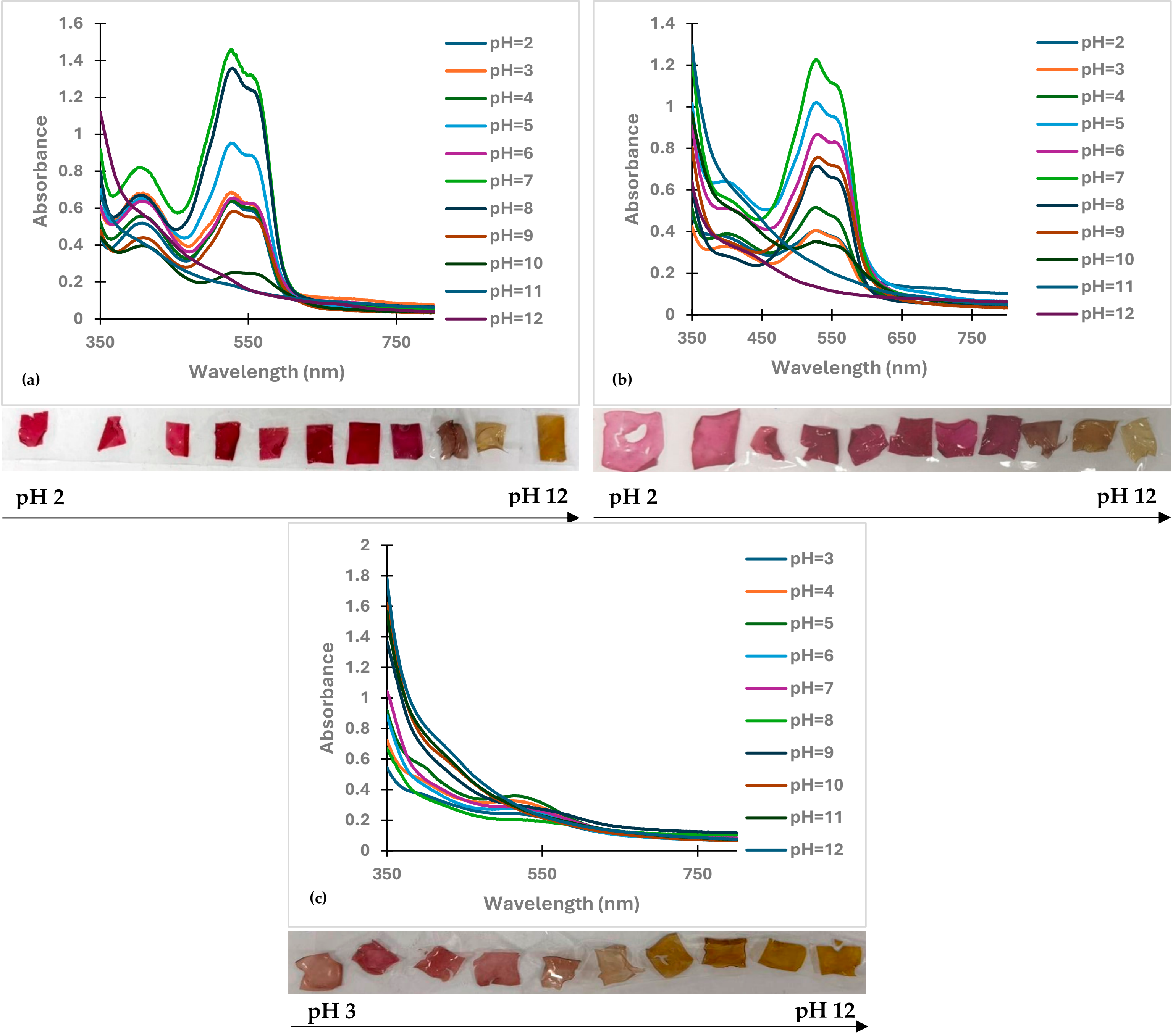

Figure S31. UV-Vis spectra of: (a) PVA-**3** film; (b) chitosan-PVA-**3** film; (c) chitosan-starch-**3** film after 2h in buffer solutions.

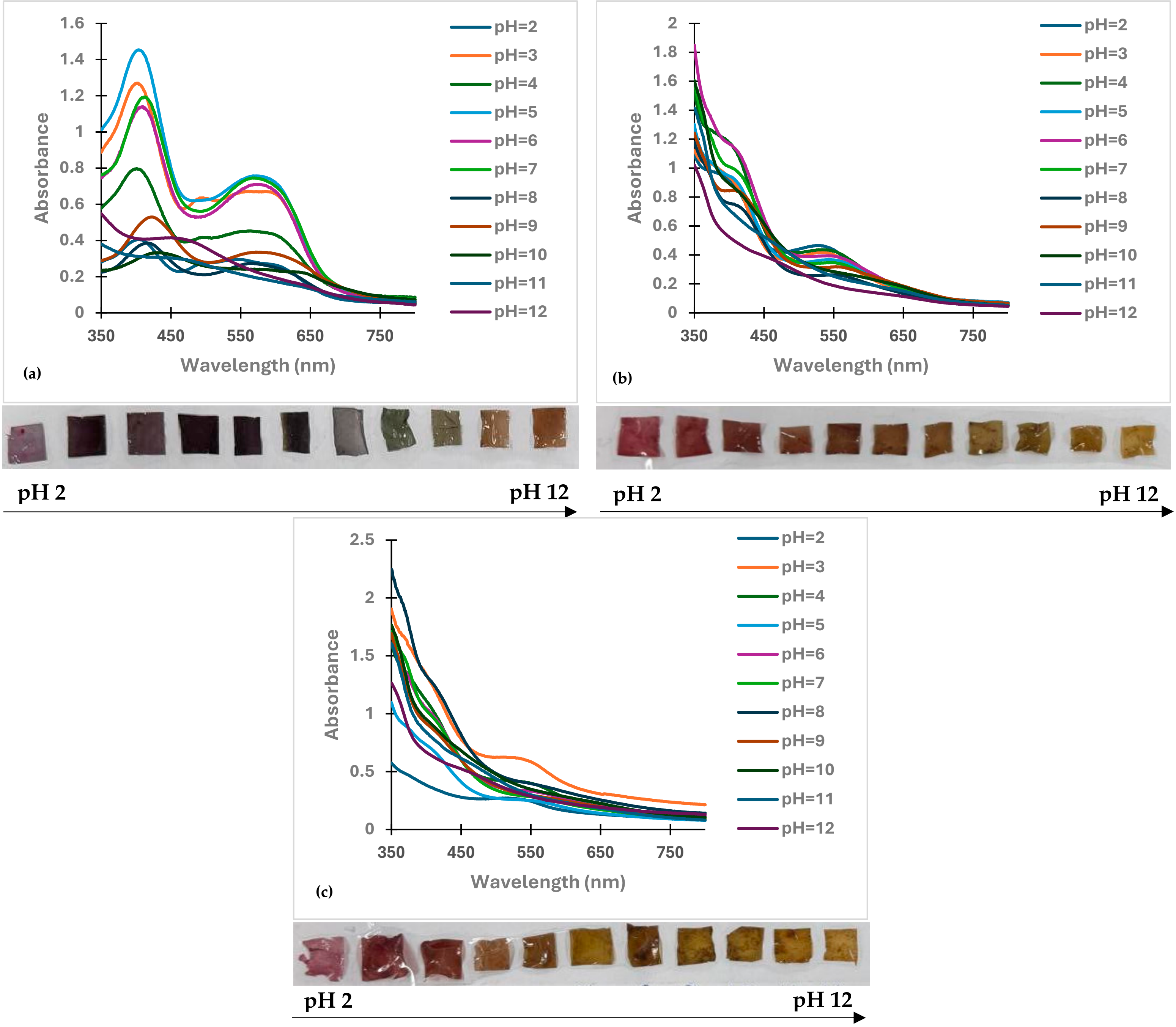

Figure S32. UV-Vis spectra of: (a) PVA-**4** film; b) chitosan-PVA-**4** film; (c) chitosan-starch-**4** film after 2h in buffer solutions.

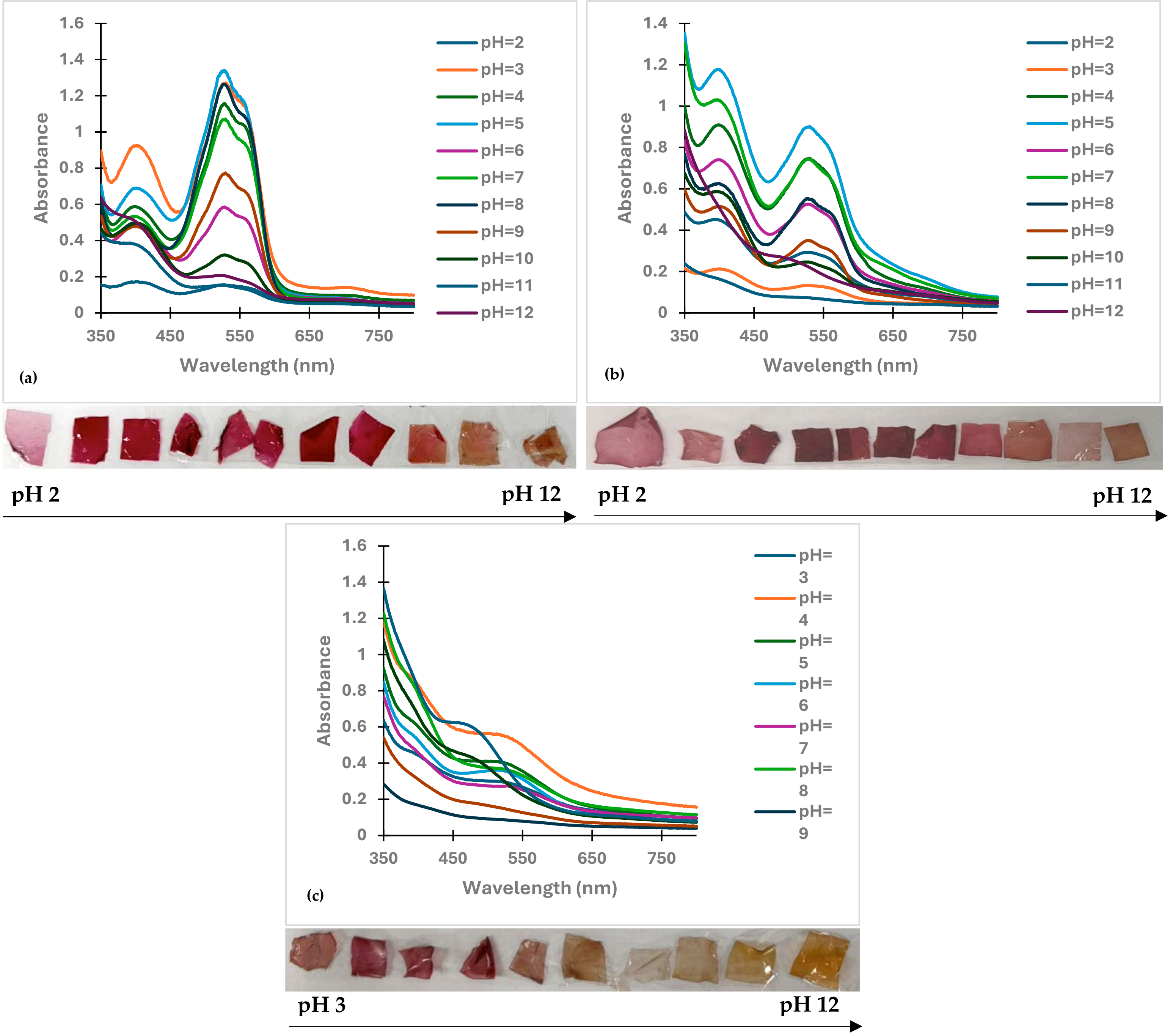

Figure S33. UV-Vis spectra of: (a) PVA-**5** film; (b) chitosan-PVA-**5** film; (c) chitosan-starch-**5** film after 2h in buffer solutions.

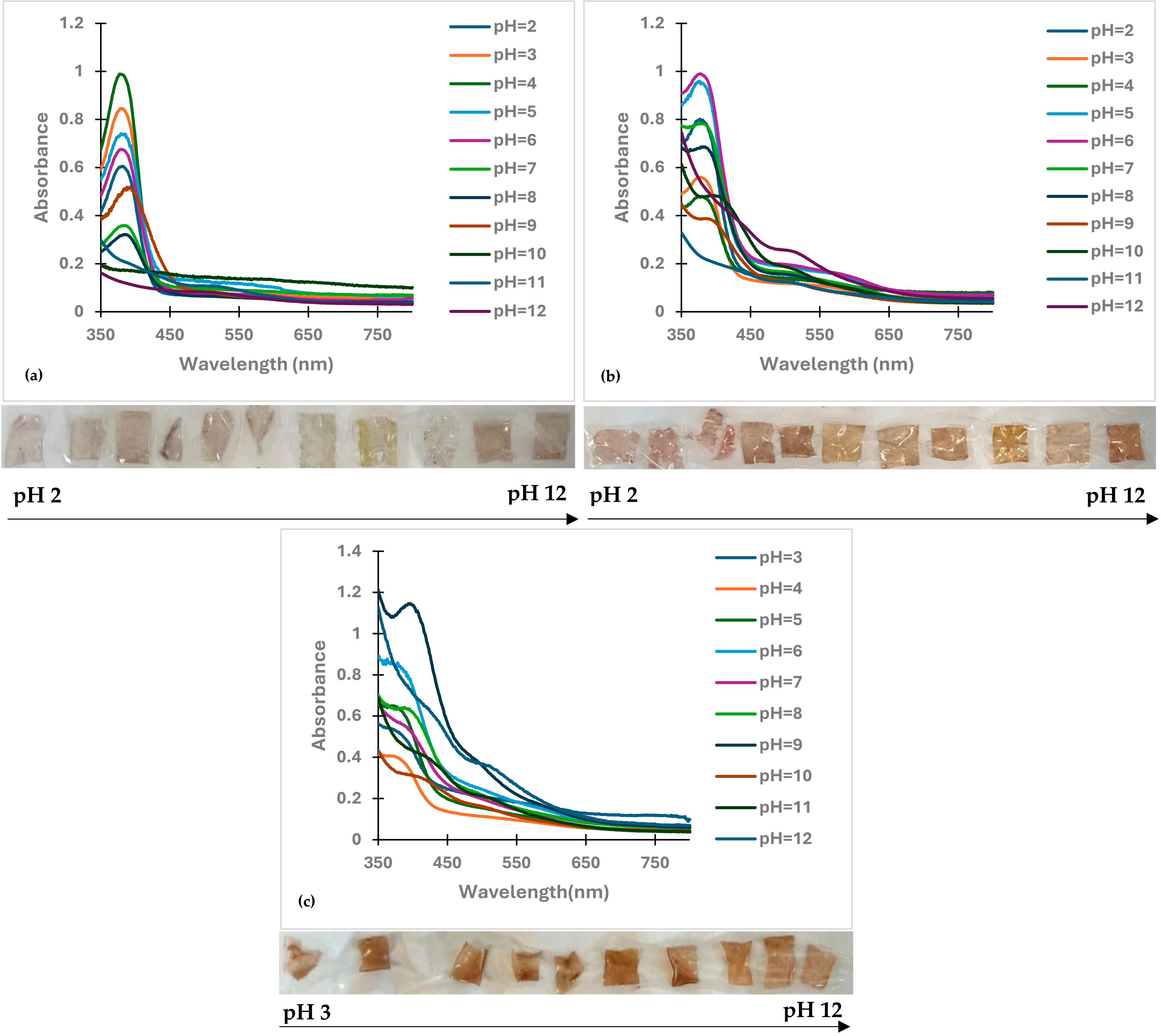

Figure S34. UV-Vis spectra of: (a) PVA-**OPE** film; (b) chitosan-PVA-**OPE** film; (c) chitosan-starch-**OPE** film after 2h in buffer solutions.

2.5. *Effectiveness of the embedded biopolymer films for monitoring the freshness of stored meat samples*

Table S7. Color changes of **compound 1** incorporated films tested on meat at 4°C, 20°C, and 40°C.

Meat	Film	Storage condition	Temperature (°C)		
			4	20	40
Pork	PVA-1	atm contact	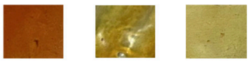		
	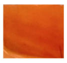	direct contact	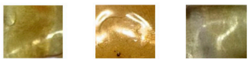		
	CHIT_PVA-1	atm contact	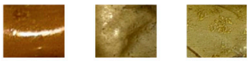		
	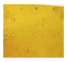	direct contact	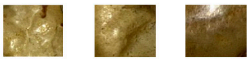		
	CHIT_STARCH-1	atm contact	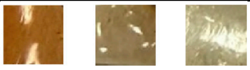		
	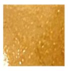	direct contact	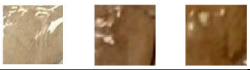		
Fish	PVA-1	atm contact	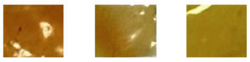		
	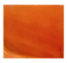	direct contact	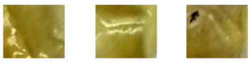		
	CHIT_PVA-1	atm contact	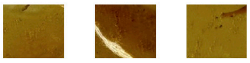		
	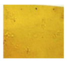	direct contact	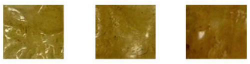		
	CHIT_STARCH-1	atm contact	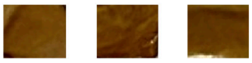		
	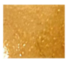	direct contact	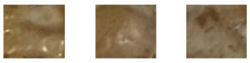		

Table S8. Color changes of **compound 3** incorporated films tested on meat at 4℃, 20℃, and 40℃.

Meat	Film	Storage condition	Temperature (℃)		
			4	20	40
Pork	PVA-3	atm contact	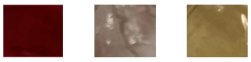		
	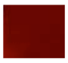	direct contact	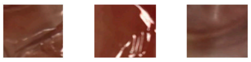		
	CHIT_PVA-3	atm contact	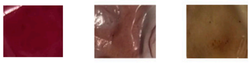		
	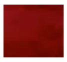	direct contact	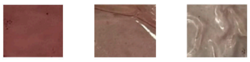		
	CHIT_STARCH-3	atm contact	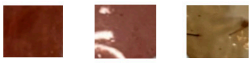		
	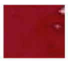	direct contact	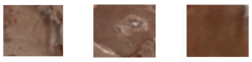		
Fish	PVA-3	atm contact	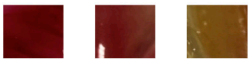		
	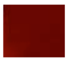	direct contact	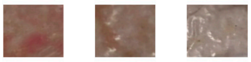		
	CHIT_PVA-3	atm contact	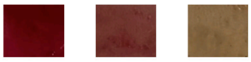		
	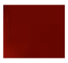	direct contact	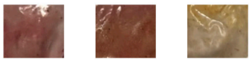		
	CHIT_STARCH-3	atm contact	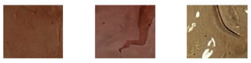		
		direct contact	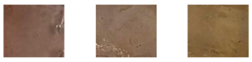		

Table S9. Color changes of **compound 4** incorporated films tested on meat at 4℃, 20℃, and 40℃.

Meat	Film	Storage condition	Temperature (℃)		
			4	20	40
Pork	PVA-4	atm contact	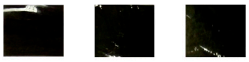		
	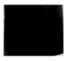	direct contact	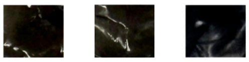		
	CHIT_PVA-4	atm contact	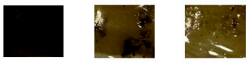		
	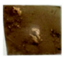	direct contact	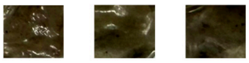		
	CHIT_STARCH-4	atm contact	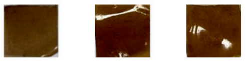		
	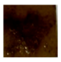	direct contact	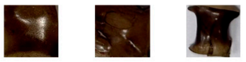		
Fish	PVA-4	atm contact	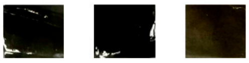		
	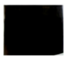	direct contact	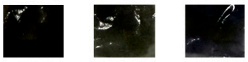		
	CHIT_PVA-4	atm contact	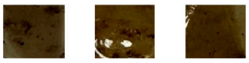		
	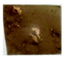	direct contact	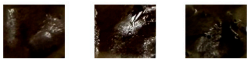		
	CHIT_STARCH-4	atm contact	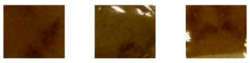		
	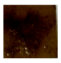	direct contact	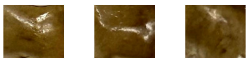		

Table S10. Color changes of **compound 5** incorporated films tested on meat at 4°C, 20°C, and 40°C.

Meat	Film	Storage condition	Temperature (°C)		
			4	20	40
Pork	PVA-5	atm contact	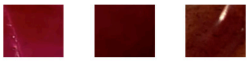		
	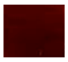	direct contact	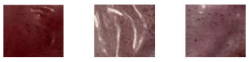		
	CHIT_PVA-5	atm contact	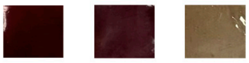		
	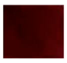	direct contact	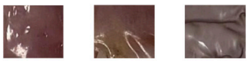		
	CHIT_STARCH-5	atm contact	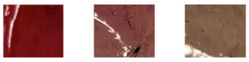		
	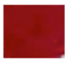	direct contact	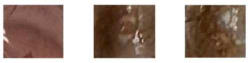		
Fish	PVA-5	atm contact	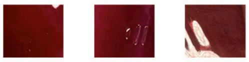		
	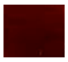	direct contact	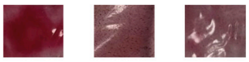		
	CHIT_PVA-5	atm contact	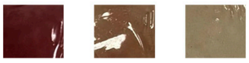		
	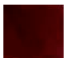	direct contact	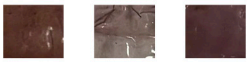		
	CHIT_STARCH-5	atm contact	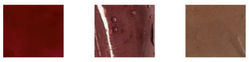		
	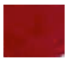	direct contact	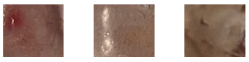		
